# Protein Dynamics of the HIF-2α PAS-B Domain upon Heterodimerization and Ligand Binding

**DOI:** 10.1371/journal.pone.0094986

**Published:** 2014-04-15

**Authors:** Matteo Masetti, Federico Falchi, Maurizio Recanatini

**Affiliations:** Department of Pharmacy and Biotechnology, Alma Mater Studiorum–Università di Bologna, Bologna, Italy; University of South Florida College of Medicine, United States of America

## Abstract

Hypoxia-Inducible Factor (HIF) transcription factors are heterodimeric proteins involved in the regulation of oxygen homeostatis. Their upregulation has been related to several tumors with a remarkably poor clinical outcome. The recent discovery of a druggable cavity in the HIF-2α PAS-B domain has opened an unprecedented opportunity for targeting the HIF-2α transcription factor in view of pharmaceutical strategies. Coincidentally, a novel compound able to selectively disrupt the HIF heterodimerization with a submicromolar activity has been reported. In this work, we investigated the molecular mechanisms responsible for the inhibition by comparing the dynamical features of the HIF-2α PAS-B monomer and the HIF-2α PAS-B/HIF-1β PAS-B complex, in the ligand-bound and -unbound states. Plain and biased Molecular Dynamics were used to characterize the differential conformational changes both structurally and energetically.

## Introduction

In human cells, oxygen homeostasis is primarily regulated by the functionality of the Hypoxia-Inducible Factor (HIF) transcription factors [1]. The transcriptionally active form of HIFs exists in a heterodimeric complex constituted by an oxygen-labile α subunit (HIF-α) and a stable β subunit (HIF-1β, also known as ARNT) [2]. Under normoxic conditions, HIF-α is constitutively downregulated mainly by proteasomal degradation. In case of low oxygen concentration, the downregulatory mechanisms are relieved, and the increased stability of the α subunit leads to an augmented transcriptional activity of the HIF complex. The response to hypoxia is eventually achieved by the expression of genes which adapt the energetic metabolism to the reduced oxygen availability and promote oxygen transport through angiogenesis and maturation of red blood cells [1], [3].

Among the three possible α subunit isoforms, HIF-1α and HIF-2α are major responders to hypoxia [3]. Since the overexpression of these subunits has been related to a number of highly malignant tumors, HIFs have recently started to be regarded as pharmaceutically relevant putative anticancer target [4], [5].

**Figure 1:**
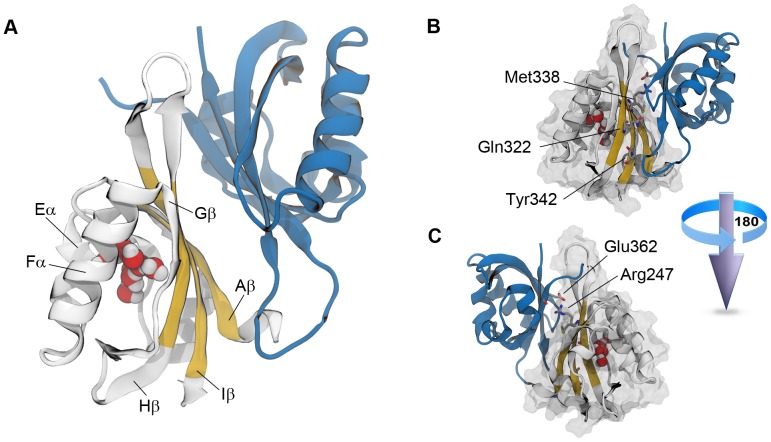
The HIF-2α PAS-B/HIF-1β PAS-B complex. A. The HIF-1β PAS-B domain is shown as blue ribbons, whereas the HIF-2α PAS-B is colored in white except for the three central β-strands of the β-sheet surface (Aβ, Iβ, and Hβ strands, in yellow). The eight crystallographic water molecules are also shown as van der Waals spheres. B. Details on important aminoacids at the interface between domains. In particular, aminoacids involved in heterodimerization (Gln322, Met338, and Tyr342) and retro-mutated aminoacids (Arg247 and Glu362) are shown as sticks. The HIF-2α PAS-B Connolly surface is shown in transparent.

From a structural standpoint, the HIF heterodimer belongs to the family of basic-helix-loop-helix Per-ARNT-Sim (bHLH-PAS) transcription factors [6]. Both α and β subunits contain an N-terminal binding domain (bHLH) and two tandem PAS domains (PAS-A and PAS-B) responsible for the dimerization process which leads to the transcriptionally active complex [7]. As revealed by both crystallography experiments [8] and NMR solution structures [9], the PAS-B/PAS-B dimerization occurs via an antiparallel interaction of the β-sheets belonging to each domain (Figure 1A). HIF-1α and HIF-2α, also carry a C-terminal regulatory sequence that interacts with coactivators of gene expression [3].

It has been shown that the HIF heterodimerization, and in turn its transcriptional activity, can be effectively hampered by specific point mutations on the solvent exposed surface of the β-sheet belonging to the HIF-α PAS-B domain (Figure 1B) [9], [10]. This finding highlights the pivotal role played by the interaction of the two PAS-B domains in the stability of the full-length transcription factor. On the one hand, the possibility of preventing the heterodimerization represents a potential opportunity to target HIF for treating tumors. On the other hand, attempting to disrupt heterodimerization with small molecules by directly exploiting the β-sheet interface of the PAS-B domains poses severe pharmaceutical challenges, both in terms of efficacy and selectivity, that strongly limit the feasibility of this strategy [11]–[13]. Recently, the discovery of a druggable preformed cavity in the HIF-2α PAS-B domain has opened a novel pharmaceutical route to target the HIF transcription factor [8]. The underlying idea of this approach is to modulate the affinity between the two domains by exploiting a ligand-induced conformational change in the HIF-2α PAS-B domain (allosteric modulation). This inhibitory strategy has been firstly advanced [8] and later validated through biophysical characterizations [14], [15] by Scheuermann and coworkers. Besides, the practical viability of the approach has also been confirmed by the discovery of a compound showing a submicromolar disrupting activity (IC_50_ = 0.1 µM, compound **32** according to the nomenclature of the original paper, see Figure S1A in File S1) [16].

In spite of these remarkable results, the recently reported crystal structure of a high affinity mutant heterodimer (HIF-2α PAS-B R247E/HIF-1β PAS-B E362R) bound to compound **32** (PDB code: 4GHI [15], Figure S1B in File S1) was similar to its apo form (3F1P [8], Cα RMSD lower than 0.3 Å). This finding makes the above reported allosteric mechanism difficult to be explained from a static point of view, calling for an in depth investigation of the dynamical behavior of these complexes.

Here, by using Molecular Dynamics (MD) simulations, we investigated the conformational behavior of the wild type HIF-2α PAS-B domain and characterized the changes in its dynamic upon binding with HIF-1β PAS-B and compound **32**, which was taken here as a prototypical disrupting ligand. Moreover, the water dynamics of the HIF-2α druggable cavity was also investigated, as it is closely related to the dynamical behavior of the protein. As a main result of this work, we show that the conformational changes responsible for the disrupting effect can be described in terms of twisting and bending deformations of the HIF-2α β-sheet surface. According to our simulations, such an effect is not caused by an allosteric mechanism in the strict sense, but can be related to a ligand-induced decreased ability of the HIF-2α β-sheet to optimally adapt to the HIF-1β counterpart. We substantiated our model of binding using biased MD simulations, and we estimated that the binding of compound **32** decreases the heterodimerization free energy of about 3–4 kcal mol^−1^.

## Methods

### Preparation of the Models and Nomenclature

The wild type HIF-2α and the HIF-2α PAS-B/HIF-1β PAS-B complex were studied without or with compound **32** bound to them. 3F1P and 4GHI were used as initial models for apo and holo forms, respectively [8], [15].

For all the systems, the HIF-2α PAS-B domain was modeled by considering residues ranging from the aminoacidic positions 239 to 346, whereas positions 358 to 465 were used to describe the HIF-1β PAS-B domain. Since 3F1P and 4GHI are crystal structures of a high affinity mutant heterodimer (HIF-2α PAS-B R247E/HIF-1β PAS-B E362R), the wild type forms were reconstructed by retro-mutating aminoacid 247 and 362 by using the Schrödinger suite of programs [17] (Figure 1B). ACE and NME capping were added at the N- and C-terminus of the proteins, respectively. All the aminoacids were considered in their standard protonation and tautomeric forms at physiological pH, with the exception of His248 (HIF-2α PAS-B) and His367 (HIF-1β PAS-B) which were modeled in the N^δ^–protonated state, as this configuration was predicted by Schrödinger to be the most favorable [17].

We stress that with the terms *apo* and *holo* form we refer to the binding state of the HIF-2α internal cavity, and exclusively with respect to compound **32**. In addition, the binding state of the same protein to the HIF-1β counterpart is referred to as *monomeric* or *dimeric* form throughout the text. Thus, for sake of clarity, we denote the four systems, as well as the corresponding simulations, according to the HIF-2α binding state with the following notations: *A* (apo-monomeric), A* (holo-monomeric), *AB* (apo-dimeric), and A*B (holo-dimeric).

### Plain Molecular Dynamics simulations

Unbiased MD simulations were performed with AMBER12 [18] running on a NVIDIA Tesla C2050 GPU system with the *pmemd.MPI* module [19], [20]. The SPFP mixed precision model was employed throughout [21].

The amber99SB-ILDN force field was used to describe the protein [22]. Compound **32** was treated with the GAFF force field [23] together with partial charged derived through the RESP procedure [24], [25] from the electrostatic potential calculated at the HF/6-31G(d)//HF/6-31G(d) level of theory with the Gaussian03 package [26]. All systems were simulated in a cubic box filled with TIP3P water model molecules [27], keeping a margin of at least 10 Å between the wells of the cell and the solute in each dimension. All the crystallographic water molecules were preserved during simulations. An occupancy of 8 water molecules was found in the internal cavity at the beginning of simulations for systems *A* and *AB*, in accordance to the corresponding crystal structure (3F1P). Periodic boundary conditions were applied in all dimensions, and the electroneutrality of the systems was reached by adding counterions.

Langevin dynamics was performed using a timestep of 2 fs together with a frictional coefficient of 5 ps^−1^ at the target temperature of 300 K. Production runs were performed in the NPT statistical ensemble by using the Berendsen algorithm under isotropic scaling at the nominal pressure of 1 bar and with a relaxation time of 2 ps [28]. Bonds involving hydrogen atoms were restrained to their equilibrium values with the SHAKE algorithm [29]. A short-range cutoff of 12 Å was used in computing the non-bonded interactions, and the neighbor list was updated each 10 integration steps. Long-range electrostatic was treated using the Particle-Mesh Ewald method [30], [31] with a grid spacing of approximately 1 Å in all dimensions, and a fourth-order spline interpolation scheme.

The systems were gradually heated to the target temperature of 300 K during 300 ps of MD in the canonical ensemble. Then we switched to the isothermal-isobaric ensemble, and the systems were equilibrated for further 200 ps. During these preliminary simulation stages, positional restraints acting on the Cα atoms of the systems were gently released, while production runs were performed on fully unrestrained systems. Systems *A* and *AB* were simulated for a total time of 500 ns each, whereas 100 ns of production was accumulated for systems A* and A*B. Coordinates were saved each 5 ps.

### Structural Analysis

The Root Mean Squared Displacement (RMSD) of atomic positions and Root Mean Squared Fluctuations (RMSF) of protein backbone were calculated after least squares fitting with the *ptraj* module of Amber12 [18]. Distances, angles, and the folding degree of α-helices were monitored with PLUMED-1.3 [32]. In particular, the latter measure was computed as:
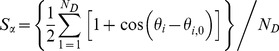
(1)where *θ_i_* is the *i* ^th^ dihedral, and *θ_i_*_,0_ was set equal to 64 and 41 degrees for *φ* and *ψ* backbone angles, respectively, in order to match the geometry of an ideal α helix. The measure is normalized over the total number of dihedral angles *N_D_*, so as to return a value of 1 in case of a completely folded helix, and zero otherwise.

### Analysis of Correlated Protein Motions

Correlated motions in the HIF-2α PAS-B domain were calculated by diagonalizing the covariance matrix of positional deviations (Principal Component Analysis, PCA), whose elements *c_ij_* are expressed as [33]:
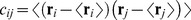
(2)where **r** *_i_* and **r** *_j_* represent the positions of atoms *i* and *j* respectively, and angle brackets denote ensemble averages. The analysis was performed for systems *A* and *AB* after least squares fitting to the initial structure of the HIF-2α PAS-B domain. Only Cα atoms were used for both the superimposion procedure and calculation of the covariance matrix.

The relevance of the largest fluctuation amplitude eigenvectors (here defined as the set of eigenvectors explaining 80% of the total variance, i.e. the first 20 eigenvectors) was calculated using the normalized overlap [34]:
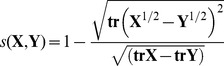
(3)were **X** and **Y** are two symmetric and diagonalized covariance matrices, and **tr** stands for the trace of the matrix. This measure returns a value of 1 when the two matrices are identical, and 0 when they are orthogonal. Here, the overlap was evaluated between different chunks of the same trajectory. Each pair of chunks differed in 100 ns of sampling size, and the overlap was stepwise evaluated by increasing the chunk size of the same amount while moving along the trajectory. Thus, over a 500 ns long simulation, four normalized overlap measures were obtained for the *A* and *AB* systems: (1/5)/(2/5), (2/5)/(3/5), (3/5)/(4/5), and (4/5)/(5/5).

The accumulation and diagonalization of the covariance matrices as well as the calculation of the normalized overlap was performed with GROMACS-3.2.1 [35].

To better capture functional correlated motions in protein dynamics, Full Correlation Analysis (FCA) [36] was performed for systems *A* and *AB* using the *g_fca* tool (version 1.3) running within the GROMACS-3.2.1 framework [35]. Resting on an information theory framework, FCA overcomes the limitations of conventional approaches such as a covariance matrix PCA. With this method, the ensemble averaged deviation from an uncorrelated distribution of random variables is given by the mutual information [36], [37]:
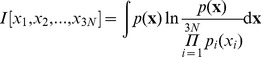
(4)where (*x*_1_, *x*_2_, …, *x*_3N_) are the components of the deviation vector **x** = **r** − <**r**>, and *p*(**x**) is the joint probability distribution which is equal to the product of all the marginal distributions *p*_i_(*x*_i_) only for totally independent random variables. In this case, the argument of the logarithm is one, and the integral vanishes returning a null mutual information. In any other case, linear, non-linear, and high-order correlation is detected, yielding a mutual information value greater than zero. In particular, the FCA method searches for the orthonormal coordinate transformation in the Cartesian space of the positional deviation vectors, by minimizing the mutual information measure. As a result, a set of maximally uncoupled linear generalized coordinate with better anharmonic features than PCA eigenvectors is obtained [36]. The *g_fca* tool implementation constructs the generalized coordinates iteratively, by using PCA eigenvectors as initial guess for the coordinate transformation. In this work, FCA was performed on the subspace defined by the first 20 PCA eigenvectors. The FCA vectors were ranked both by their fluctuation amplitude (as in PCA), and by their anharmonicity, which is defined as the difference in the observed density and that of a normal distribution with the same variance [36].

### Water Egress Channels and Water Relaxation Time

Water egress form the HIF-2α PAS-B internal cavity was monitored for systems *A* and *AB*. For both systems, the principal inertia axes of HIF-2α PAS-B domain (calculated over Cα atoms of the initial structures) were centered and aligned along the Cartesian axes. The configurations of the trajectories were then aligned to this reference frame by least squares fitting with the *ptraj* module of Amber12 [18]. Since the cavity of the HIF-2α PAS-B domain is quite buried, and bulk water molecules can rapidly enter and exit without reaching the deepest portions of the pocket (unproductive water ingresses events), to better trace effective water channels inside the protein, a differential definition of the cavity was employed. Accordingly, the cavity was described by a couple of spheres centered at the coordinates (+2, −1, 0) with a radius of 5 and 12 Å. The smaller sphere accounted for the deepest moiety of the pocket, whereas the shell between the two spheres described the entrance of the cavity (Figure S2 in File S1). Water egress channels were then traced by monitoring the trajectories of all water residues reaching the internal sphere.

The relaxation time of water molecules inside the cavity was calculated using a “shell” survival time autocorrelation function *C_r_*(*t*) [38], [39]:
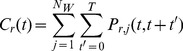
(5)where the survival function *P_r_*_,*j*_ is a step function taking a value of 1 if the *j* ^th^ water molecule is located in a shell of radius *r* from time *t* to (*t*+*t*’), and zero otherwise. In the summations, *N_w_* is the total number of water molecules whereas *T* is the total simulation time. The autocorrelation function was calculated for a shell radius *r* = 12 Å, accordingly to the above reported definition of cavity, and normalized to the value of *C_r_*(0). The water escape relaxation time *τ*, which represents the average water residence time in the cavity, was calculated by fitting both a single- and a double-exponential function to the *C_r_*(*t*)/*C_r_*(0) decay function [38]–[40].

The analysis of water molecules was performed with in house Tcl scripting running within the VMD-1.9 visualization program [41].

### Biased Molecular Dynamics simulations

An elastic network model (ENM) [42] of the Cα atoms belonging to the HIF-2α β-sheet was built with the *elNémo* web server [43] using a distance cutoff of 8 Å (Figure S3 in File S1). To better quantify the flexibility features of the β-sheet, only strands of same length and geometry should be considered [44]. To this aim, only the three central strands with 6 residues per strand of the entire β-sheet surface were used in the ENM definition (Aβ strand: residue 243 to 248, Hβ strand: residue 320 to 325, Iβ strand: residue 337 to 342). The two lowest frequency eigenvectors were used as collective coordinates to describe the β-sheet flexibility in terms of “twisting” (eigenvector 1) and “bending” (eigenvector 2) modes [44].

Umbrella sampling (US) simulations [45], [46] were performed in the NVT ensemble using the NAMD-2.8 program [47] plugged with PLUMED-1.3 [32] along the previously described low dimensionality space for all the four systems. The same force fields parameters and simulation conditions utilized for unbiased MD simulations were used. The collective coordinate space was sampled in the range of [-2.5∶2.5] Å in each dimension using a grid spacing of 0.5 Å, for a total of 121 windows. A force constant of 50.0 kcal mol^−1^ Å^−2^ was used for both the coordinates. Each window was simulated for 600 ps, where the first 100 ps served as equilibration, and the remaining 500 ps for sampling purposes. The unbiased potential of mean force (PMF) was calculated using Grossfiled’s implementation [48] of the weighted histogram analysis method (WHAM) [46], [49]. The US convergence was assessed by computing the free energy difference between the PMF obtained using the first half and the whole sampling of each window.

### Mapping the Free Energy Profiles

To compare the effect of heterodimerization and ligand binding on the flexibility of the HIF-2α β-sheet, a dynamic energy landscape approach was adopted [50]. In analogy with Marcus theory of electron transfer [51], protein conformational transitions are described by as many reduced dimensionality energy landscapes as bound and unbound states are conceived [52]–[54]. In this specific case, the perturbation on the β-sheet surface upon heterodimerization was modeled as a switching between two surfaces representing the protein conformational free energy in the monomeric and dimeric states. Two pairs of surfaces are therefore envisioned whether ligand bound and unbound states are also considered. For convenience, the perturbation of the β-sheet surface was described in terms of lowest free energy profile along the twisting coordinate only, as this mode of motion turned out to be the most informative in highlighting structural differences among the four considered system.

The energy minimum of the free energy surface for system *A* (located at the twisting coordinate here defined as *x* ^0^ *_A_*) was used as reference to map the three remaining curves corresponding to systems *AB*, A*, and A*B (Figure S4 in File S1). Each curve represented the free energy change along the most relevant conformational coordinate, whereas the energy difference between minima is a measure of the standard binding free energy ΔG°_bind_ between the considered partners, that is *A* versus *AB*, and A* versus A*B (protein-protein association free energy). Here, we are interested in obtaining an estimate of the change in heterodimerization free energy difference (ΔΔG°_bind_) upon binding of compound **32** by simple geometric considerations. To this aim, we assume the protein-protein association free energy to be composed by a vertical gap contribution (ΔG°_vert_), which describes the free energy of association between the considered partners as if they were rigid bodies, and a relaxation contribution (ΔG_relax_), always favorable, arising from the mutual conformational adaptation upon binding:
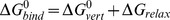
(6)


With these definitions in place, it is possible to plot the free energy curves for systems *A* and *AB*, only if we attribute to ΔG°_bind,*A*/*AB*_ a given undetermined (negative) value (Figure S4, upper panel, in File S1). The advantage of such a construction is that now we can draw the remaining plots for the A* and A*B systems without any further assumptions. Thus, the minimum of the A* energy profile, *x* ^0^ _A*_, is matched with curve *A*, where the offset between the two curves can be interpreted as the perturbation of the ligand on the protein conformation (i.e. the strain energy required by the *A* apo form to achieve the same amount of perturbation provided by ligand binding). Similarly, the A*B profile is matched with that of *AB* in *x* ^0^ _A*_. This can be explained by considering that the vertical gap (i.e. the rigid-body association free energy component) in correspondence of this value of the twisting coordinate for the A*/A*B system must be equal to the *A*/*AB* vertical gap in *x* ^0^ _A*_, as the same conformations of the two partners are considered. By geometrical considerations, the change in protein-protein association free energy due to the presence of the ligand (ΔΔG_bind_) can be eventually estimated relatively to ΔG°_bind,*A*/*AB*_ by taking the energy difference between the *x* ^0^ *_AB_* and *x* ^0^ _A*B_ minima (Figure S4, lower panel, in File S1).

## Results and Discussion

### Stability of the Proteins

**Figure 2:**
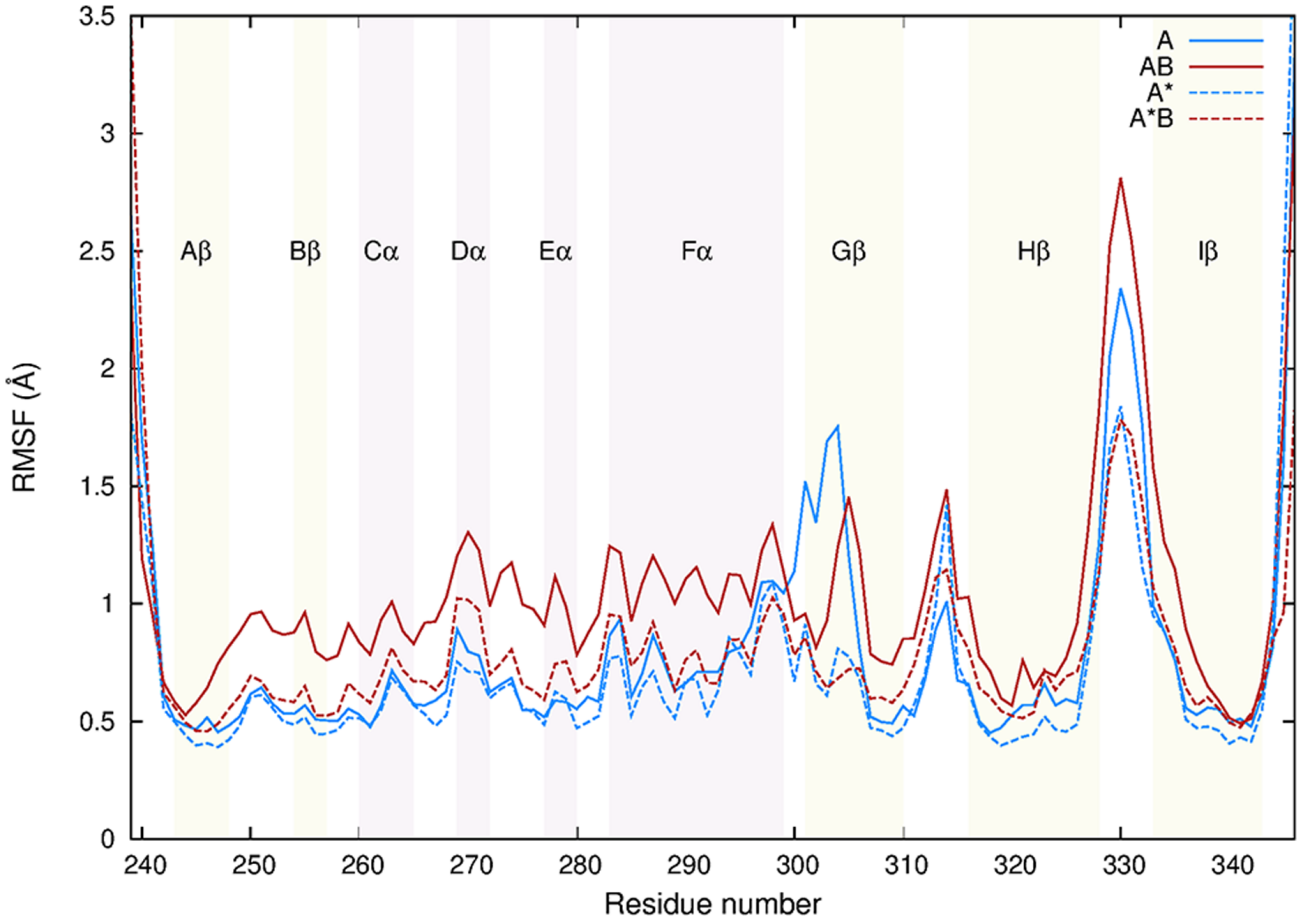
RMSF of protein backbone. Structural stability of the HIF-2α protein backbone compared between the simulated systems. For clarity, the position of the secondary structure elements are shown.

The overall stability of the proteins during unbiased MD simulations was assessed by monitoring the RMSD of Cα atoms over time (Figure S5 in File S1). Similar positional deviations were experienced by the HIF-2α PAS-B domain in the four simulated systems, showing an average RMSD of about 1.0 or 1.5 Å compared to the initial structure. To highlight the more flexible regions of the protein, the RMSF of the backbone averaged per residue and over time was calculated (Figure 2). As the plot shows, the secondary structure elements were quite stable, displaying an average RMSF of less than 1 Å in all the systems except for *AB*, where slightly larger fluctuations were observed (RMSF of about 1.2 Å in helices Dα, Eα, and Fα). An increased flexibility was also found in the Gβ strand for systems *A* and *AB* (RMSF around 1.5 Å), whereas the Hβ-Iβ loop turned out to be the most flexible portion of HIF-2α in all systems, reaching a maximum value of about 3 Å in *AB*.

The stability experienced by HIF-2α under different simulation conditions (i.e. bound/unbound with HIF-1β or compound **32**) was surprising. Indeed, in a previously reported MD investigation [14], HIF-2α has been shown to undergo large conformational transitions between two major states referred to as open and closed. In particular, the motion leading to the open state was defined as: i) a bending movement of the Fα N-terminus compared to the protein core of about 20–30 degrees, and ii) an unfolding of Dα and Eα helices, so that a 5–8 Å wide channel was formed in the front of the protein. As a whole, a difference of 2.2–3.6 Å in Cα RMSD was shown between the closed and open state, and the underlying conformational transition has been reported to be implicated in the solvation of the HIF-2α internal cavity, and in turn in ligand binding [14]. In our case, the RMSD and RMSF profiles clearly depicted a different scenario, as average deviations lower than 1 Å were observed among the systems. To better compare our results with previous work [14], we monitored the distances between Dα, Eα, Aβ-Bβ, and Fα, as well as the angles formed between Fα and Gβ (Figure S6 in File S1). These variables were chosen to detect the opening of the channel in the front of the protein and to describe the orientation of the Fα helix, respectively. Very low differences were observed between the systems. However, while for system *A* fluctuations in the monitored distances were in the order of about 1 Å, a less tight behavior was observed for system *AB*. Indeed, in this system the Eα-Fα distance decreased of about 3 Å along the trajectory, whereas the Fα helix moved away from the Gβ N-terminus of almost 5 degrees. Concerning helices Dα and Eα, the folding state of Dα was preserved throughout the simulations, while repeated one-turn folding/unfolding events were observed for Eα in both systems, with a higher occurrence frequency in system *AB* (Figure S6 in File S1). Even though the amplitudes of such motions are much smaller than those previously reported, the data point out a slight departure from initial conformation for system *AB*. In attempting to better highlight such conformational motions, and possibly to relate them with the allosteric effect responsible for the ligand-induced protein-protein disruption, a more in depth analysis of MD trajectories was undertaken.

### Correlated Protein Motions

Protein conformational transitions are often difficult to be detected by analyzing the time evolution of arbitrarily chosen degrees of freedom. In this respect, it is often useful to gather as much information as possible relying on an unsupervised description of collective modes of motions. An established way to accomplish this task is to perform PCA over Cα positional deviations sampled along the simulation [33]. However, a sufficiently converged exploration of the conformational space is a fundamental requirement to obtain meaningful results with this method [34].

PCA was performed for systems *A* and *AB* and the convergence of sampling was assessed in terms of normalized overlap. A satisfying overlap (greater than 0.9) was obtained in both systems after 400 ns of sampling, while extending further the simulation resulted in a decrease in overlap of about 0.025 units for *AB* (Figure S7 in File S1).

Rather than directly proceeding in analyzing the modes of motion, in the search for subtle and not necessarily linearly correlated movements, the FCA method [36], [37] was employed using as initial guess the reduced dimensionality space provided by the first 20 eigenvectors. Differently from PCA, where the resulting eigenvectors are solely ranked on the basis of their fluctuation amplitudes, FCA modes are additionally endowed with a measure of the anharmonicity of the corresponding motion [36]. This latter feature is especially appealing when analyzing protein dynamics, as most functional motions are thought to be in general anharmonic transitions, driven by an underlying multiple well free energy surface [33].

**Figure 3:**
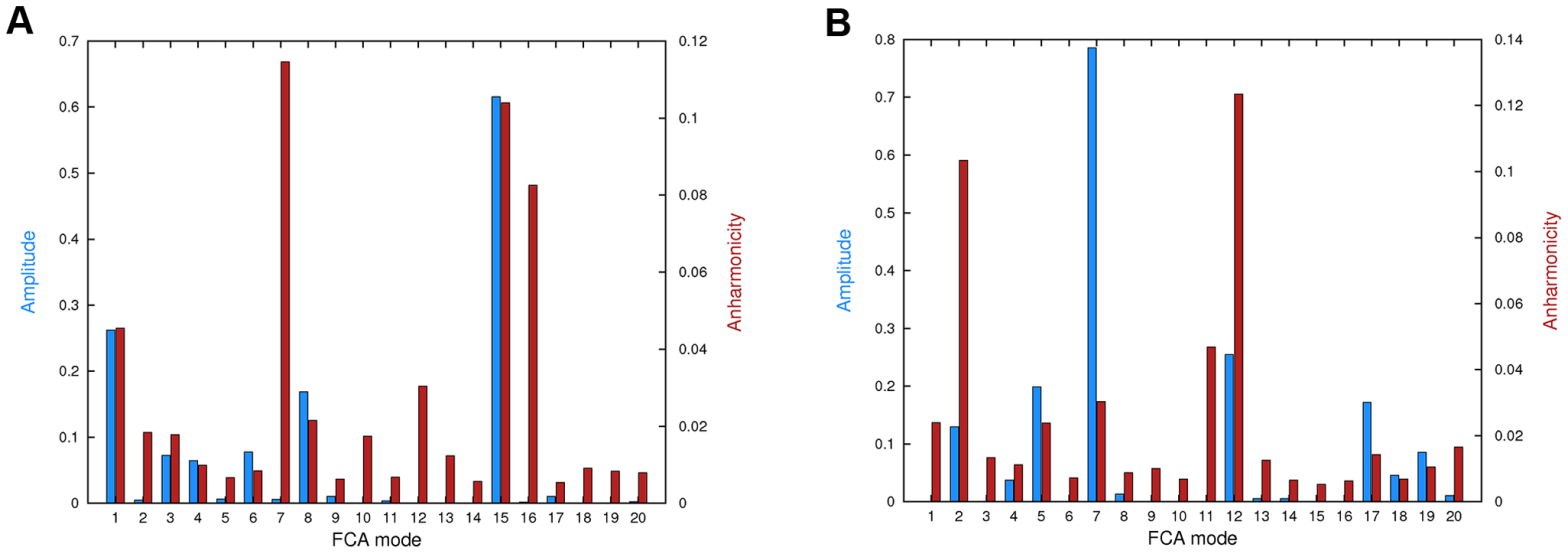
FCA modes analysis. Average fluctuation amplitude (variance, expressed in nm^2^, left *y* axis, shown as blue bars) and anharmonicity (arbitrary units, right *y* axis, shown as red bars) for the 20 FCA modes calculated in the *A* and *AB* systems (panels A and B, respectively).

In general, no correlation between amplitude and anharmonicity of motion was found, except for FCA mode 15 in system *A* that will be discussed later (Figure 3). In order to identify the essential functional motions in the HIF-2α dynamic, we focused our attention on FCA modes showing at the same time large fluctuations and anharmonic features. In this respect, the essential subspace of system *A* could be described by FCA modes 15, 1, and 8, while modes such as 7 or 16 were not considered as much informative since, despite their remarkable anharmonicity (more than 0.08 units), they lacked of an interesting amplitude of motion. Moreover, FCA mode 1 was also considered as not functionally relevant, as it involved the rigid body twisting of the Hβ-Iβ turn (Figure 1) with respect to the remainder of the protein (we note that such a mode is also present in the *AB* system at rank position 5).

**Figure 4:**
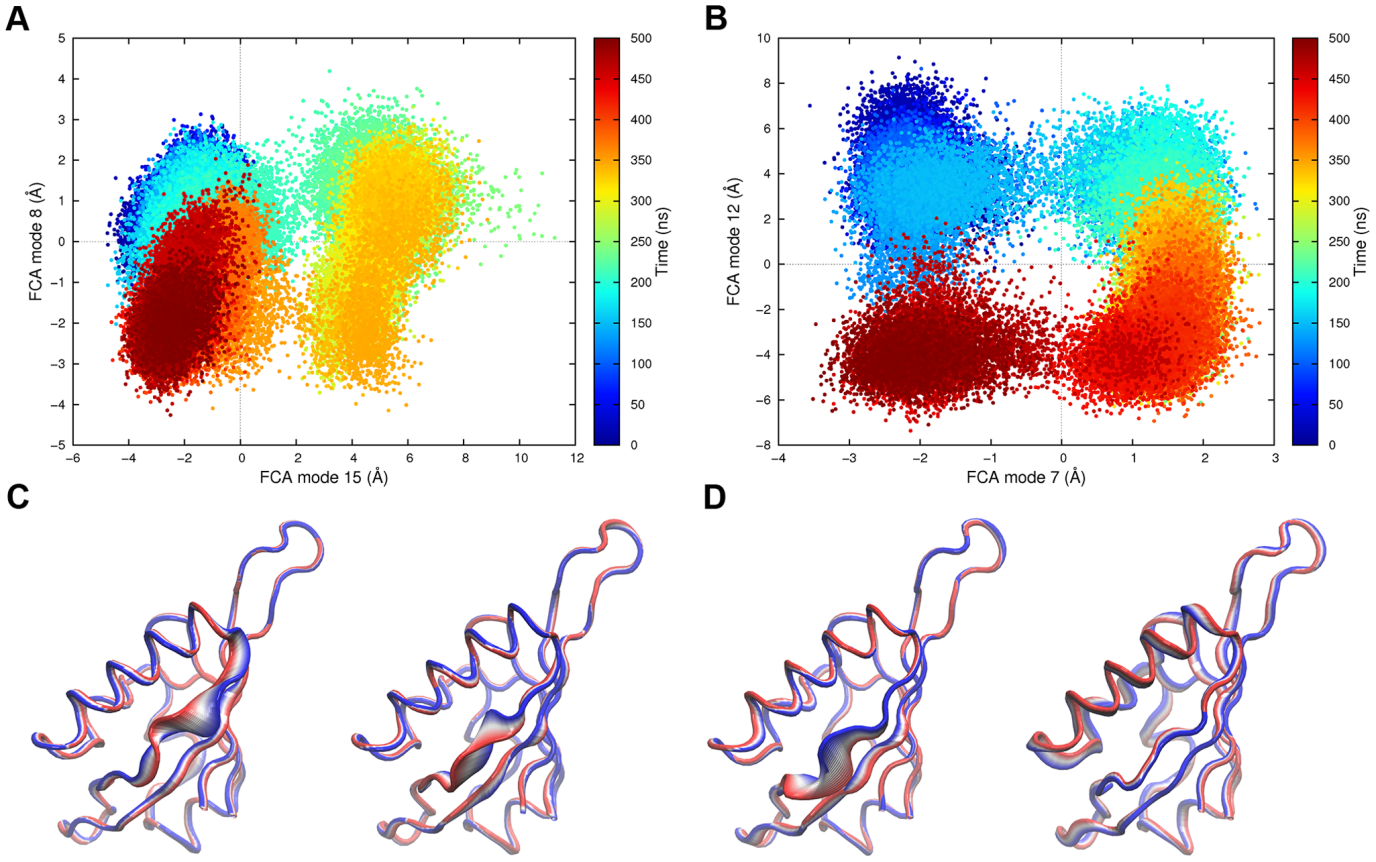
Essential FCA space. Projections of the unbiased MD trajectories along the most relevant FCA modes for system *A* and *AB* (panels A, and B. respectively). In panels C and D, a pictorial representation of the collective motions along the same FCA modes is reported for system *A* and *AB*.

Having identified the most relevant collective modes of motions for system *A*, it is important to evaluate their time evolution along the trajectory. In Figure 4, the projections of the MD trajectories along the relevant FCA subspace is shown together with a pictorial representation of the corresponding motion. As it can be seen in Figure 4A, FCA mode 15 successfully distinguished two underlying free energy wells, whereas a less clear cut separation was found along mode 8, reflecting its significantly lower anharmonicity (0.02 versus more than 0.1 units). As illustrated by Figure 4C, FCA mode 15 and 8 describe the N- and C-terminal opening of Gβ strand, spanning 7 and 4 Å, respectively. Aside from an intrinsic flexibility on the Gβ strand, these results confirmed a very stable and tightly structured protein, in line with the striking consensus found among the various crystallographic structures so far reported [8], [15]. As already mentioned, this behavior was to some extent surprising but not completely unexpected, as the lacking of a large scale plasticity could be already inferred by the PCA eigenvalues spectra, where up to 20 eigenvectors were needed to account for about 80% of the total variance in both systems. On the contrary, in case of large conformational transitions, a considerably smaller amount of eigenvectors are in general required to explain the same total variance (typically ranging from 3 to 5) [33].

A similar behavior was found for the *AB* system, with slight, but important, differences. First of all, we note that the amplitude/anharmonicity plot shown in Figure 3B is not as easily readable as that of system *A*, thus complicating the selection of the essential subspace. Here, our attention was focused on FCA modes 7 and 12, showing a remarkably large fluctuation amplitude the former, and high anharmonicity the latter. By observing the pictorial representation of mode 7 in Figure 4D, it is possible to recognize that the correlated motions mostly take place in the same region of the Gβ strand involved in mode 8 for system *A*. However, this similarity should not be overrated, as the detailed atomic motion described by the considered eigenvectors is entirely different. In spite of this, intrinsic flexibility of HIF-2α is retained upon binding to HIF-1β, which could be functionally relevant. Indeed, the Gβ strand intrinsic flexibility was found to be responsible for some aspects of the hydration features of the HIF-2α PAS-B internal cavity (see below), and it is most likely involved in ligand recognition. Concerning FCA mode 12, it involves a quite collective breathing motion that mostly affects the position of Fα helix with respect to the remainder of the protein. Finally, we note that the large variance mode 2, corresponding to a rigid-body bending motion of the Hβ-Iβ turn and that was not considered here because of its low degree of anharmonicity, roughly matched FCA mode 7 of system *A*.

Since no large scale transitions were observed for HIF-2α in a submicrosecond timescale, two major questions had to be addressed. First, what is the hydration behavior of the internal cavity and how does it change upon HIF-1β binding, and second, how can a ligand affect the PAS-B/PAS-B interaction, as ligand-induced conformational selection seems not to be conceivable.

### Cavity Hydration and Water Dynamics

Even though not directly involved with the ligand-induced disrupting effect, it is important to monitor the HIF-2α cavity hydration for system *A* and *AB* to ascertain proper water dynamics in the absence of large scale conformational motions. Moreover, the identification of water channels is useful to confirm the local flexibility of the protein.

**Figure 5:**
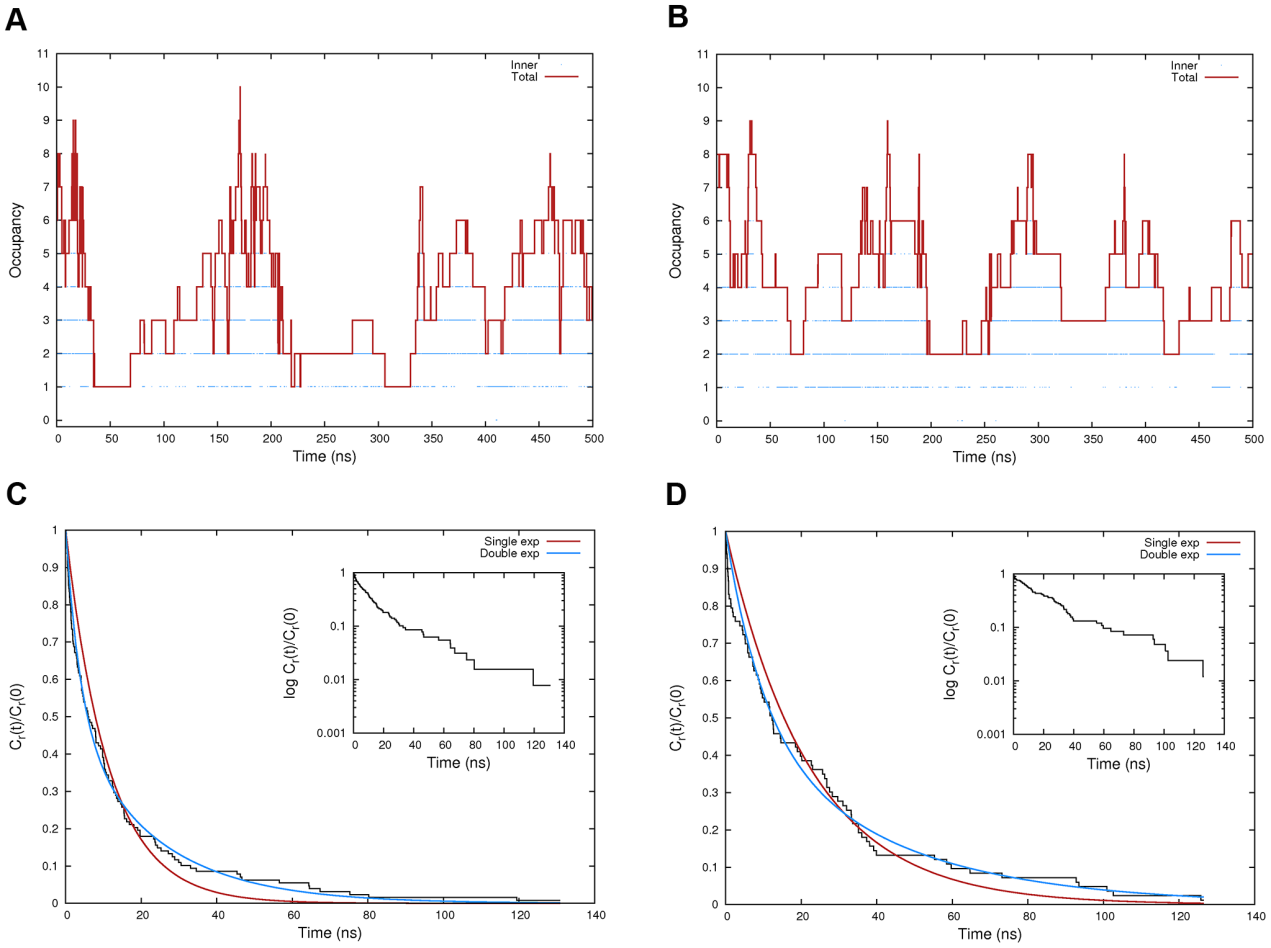
Water dynamics. Total and inner cavity water molecules occupancy (depicted as red lines and blue dots, respectively) monitored along the unbiased MD trajectory for systems *A* (panel A), and *AB* (panel B). In panels C and D, the normalized “shell” autocorrelation function is shown as a black line for both systems. The single and double exponential fitting of each curve is represented as a red and blue line, respectively. Moreover, for both C and D panels, an inset displaying the semi-logarithmic plot of each *C_r_*(*t*)/*C_r_*(0) function is also reported.

The water occupancy of the HIF-2α internal cavity was monitored over time in terms of total occupancy and the occupancy of the inner portion. As reported in Figure 5, the systems started with a total occupancy of eight water molecules, consistently with 3F1P. Over time, the total occupancy ranged from 1 to 10 for system *A* and from 2 to 9 for *AB*. Notably, the inner portion of the cavity was very seldom found to be completely dehydrated and, as shown in Figure S8 in File S1, an average occupancy of 2 water molecules was found.

Even though a differential hydration behavior could be inferred by the frequency of the fluctuations shown in Figure 5A and 5B, to characterize and quantify differences, a more detailed analysis of water dynamics was carried out. To this aim, we calculated the residence time of water molecules inside the cavity from a “shell” survival autocorrelation function (Figure 5C and 5D, black line). The plots show a somewhat slower decay of the autocorrelation function for system *AB* when compared to the HIF-2α monomer. This feature can be better appreciated by comparing the slope of the two curves in the semi-logarithmic plots reported as insets in the same Figure. The water escape relaxation time was then calculated by fitting both a single and a double exponential curve to the autocorrelation function. As it is shown in Figure 5C and 5D, the single exponential fitting was rather poor (red line), whereas a better agreement between the calculated and the analytical function could be obtained by using a double exponential (blue line). Indeed, the Root Mean Squared Error of the fitted curve reduced from 0.040 to 0.015 for system *A*, and from 0.044 to 0.025 for *AB*. This behavior clearly underlies a dual lifetime regime for water molecules, that in general can be either related to the amino acid character (charge, hydrophilic or hydrophobic) or to the curvature of the protein surface [38], [39], [55].

The water escape relaxation times calculated from the double exponential fitting were τ_fast_ = 3870 ps and τ_slow_ = 22500 ps for system *A*, and τ_fast_ = 9660 ps and τ_slow_ = 39320 ps for *AB*. It is known that the residence time of water molecules in contact with protein ranges from 10–50 ps for mobile water molecules located at the protein surface, up to nanoseconds and milliseconds for more buried interaction sites [56]. In this case, the confining effect of the protein is responsible for the moderately high relaxations times observed, whereas the dual lifetime regime can be principally attributed to the shape of the pocket. Indeed, water molecules reaching the deepest portions of the pocket will behave as kinetically distinct from those at the exterior, which more easily can escape from the cavity. The significantly higher characteristic times obtained for the heterodimer clearly indicate that, even though the HIF-1β binding has a limited effect on the water occupancy of the internal cavity, it has indeed a strong impact on their relaxation kinetics. This finding can be rationalized by hypothesizing a dimerization-induced stiffening effect exerted by HIF-1β over its α counterpart. In other words, taken together, the analyses suggest that the heterodimerization does not change considerably the overall shape of the internal cavity, but it does increase the ability to retain water molecules. Notably, this effect might also apply for ligands bound to the HIF-2α internal cavity.

**Figure 6:**
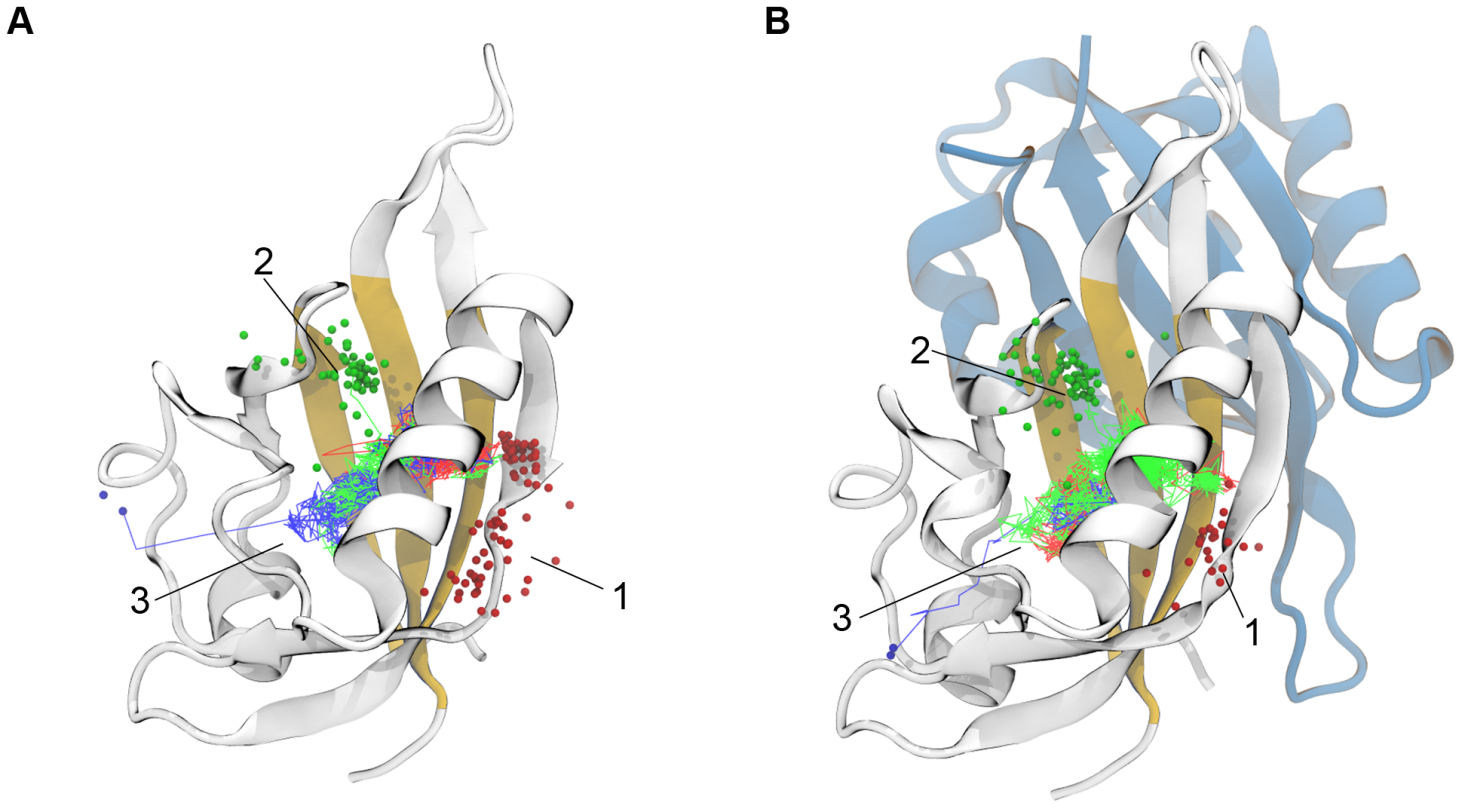
Water egress channels. The *A* and *AB* systems are shown in panels A and B, respectively. The spheres indicates the egress channel, whereas broken lines represent examples of typical escape routes along channel 1 (red), channel 2 (green), and channel 3 (blue). The protein ribbon color code follows that reported on Figure 1.

To further investigate this phenomenon, we traced the trajectory of the escaping water molecules, and by doing so we additionally characterized the preferred egress pathways (Figure 6A and 6B). For both systems we distinguished two major egress routes that, by adopting the nomenclature introduced by Scheuermann [14], we denoted as “channel 1” (located between Fα and Gβ) and “channel 2” (between Fα and Eα), plus a third winding pathway (“channel 3”) much less populated than the others by which water molecules escaped the protein in proximity of the Gβ-Hβ turn. The relative preference of the three channels were in order 61%, 37%, and 2% for system *A*, and 27%, 71%, and 2% for system *AB*. Channel 1 is further composed by two sub-channels involving either the N- or the C-terminal portion of the Gβ strand, in agreement with the intrinsic flexibility highlighted by FCA for the monomeric HIF-2α PAS-B domain (Figure 6A). Conversely, in the heterodimeric system, the C-terminal sub-channels was completely suppressed as a result of a stiffening effect induced by the interaction with the HIF-1β counterpart (Figure 6B). These results support the idea that the change in water kinetics might be related to the partial obstruction of the most accessible pathway for water egress, that is channel 1. Additionally, as expected, there is no need for large scale motions in the HIF-2α PAS-B domain to keep the internal cavity on average fully hydrated.

As already mentioned, the conformational motions experienced by the Gβ strand provide a “gate” to the interior of the protein that might also be exploited by ligands while reaching the buried binding site in the internal pocket.

### Elastic Network Analysis and Dynamical Energy Landscapes

Since no large scale conformational transitions for the apo and monomeric form of the HIF-2α PAS-B domain were found, we asked whether it was possible to link protein-protein disruption mechanism to more localized and subtle protein motions. According to the literature, it is reasonable that ligand binding to the HIF-2α internal cavity might alter the shape of the β-sheet surface in proximity of the PAS-B/PAS-B interface so as to modulate the heterodimerization [15].

**Figure 7:**
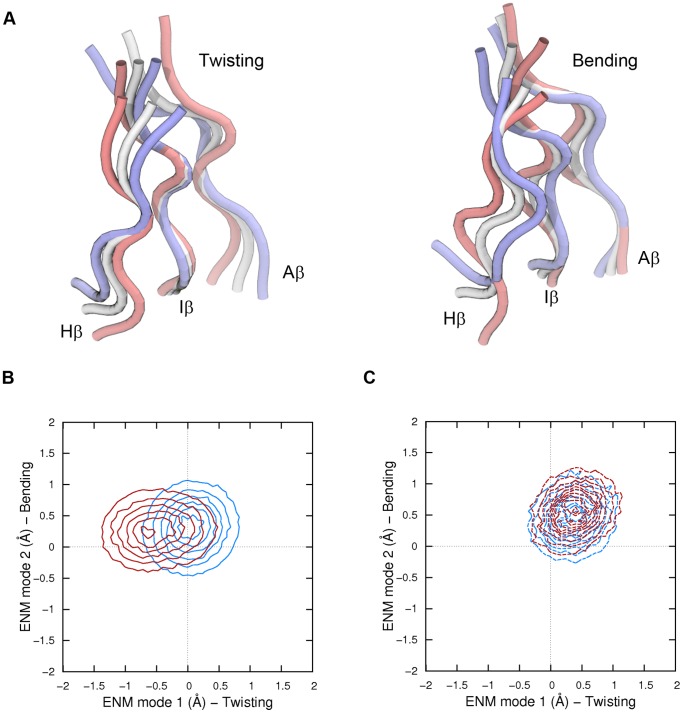
The “twisting” and “bending” modes of motion. A. Idealized β-sheet modes of motions obtained through the ENM analysis. B and C. Normalized probabilities along the twisting and bending modes of motions for systems *A* and *AB* (panel B, blue and red lines, respectively), and for systems A* and A*B (panel C, dotted blue and red lines, respectively).

Large scale conformational transitions in proteins are usually supposed to underlie “soft” mode of motions. On the contrary, by virtue of their tertiary packing, β-sheet structures are expected to experience in comparable timescales much smaller fluctuations. This means that, by borrowing the terminology of Normal Mode Analysis, in searching for dynamical differences among the simulated systems one should look for high frequency eigenvectors. To better highlight these local changes we decided to build an ENM of the β-sheet surface only (Figure S3 in File S1). In line with previous studies [44], [57], the lowest frequency modes derived by the model roughly matched a “twisting” and a “bending” motion of the surface (Figure 7A). The probability distributions of the unbiased MD trajectories projected on the space defined by the lowest frequency ENM modes (hereafter simply referred to as twisting and bending modes) are shown in Figure 7B (system *A* versus *AB*) and 7C (system A* versus A*B). The shape of the β-sheets at the beginning of the simulations was mapped on this low dimensionality space at zero values of twisting and bending. The plots clearly underline small but significant differences among the four systems, and we stress that these differences are dynamical, i.e. gained along the trajectory.

In order to substantiate the analysis with quantitative considerations, it would be tempting to calculate the PMF along the twisting and bending coordinates as a probability ratio directly from the distributions shown in Figure 7B and 7C. However, to strengthen our results, we performed brand new 2D-US simulation on the same collective coordinate space for all the systems. In Figure S9A-D in File S1 the reconstructed PMFs are shown, whereas in Figure S10A-D in File S1 we report an estimate of the statistical error related to unconvergence of sampling. As the data show, the PMFs could be considered satisfactorily converged, and the overall picture drawn by the unbiased MD simulations was confirmed.

**Figure 8:**
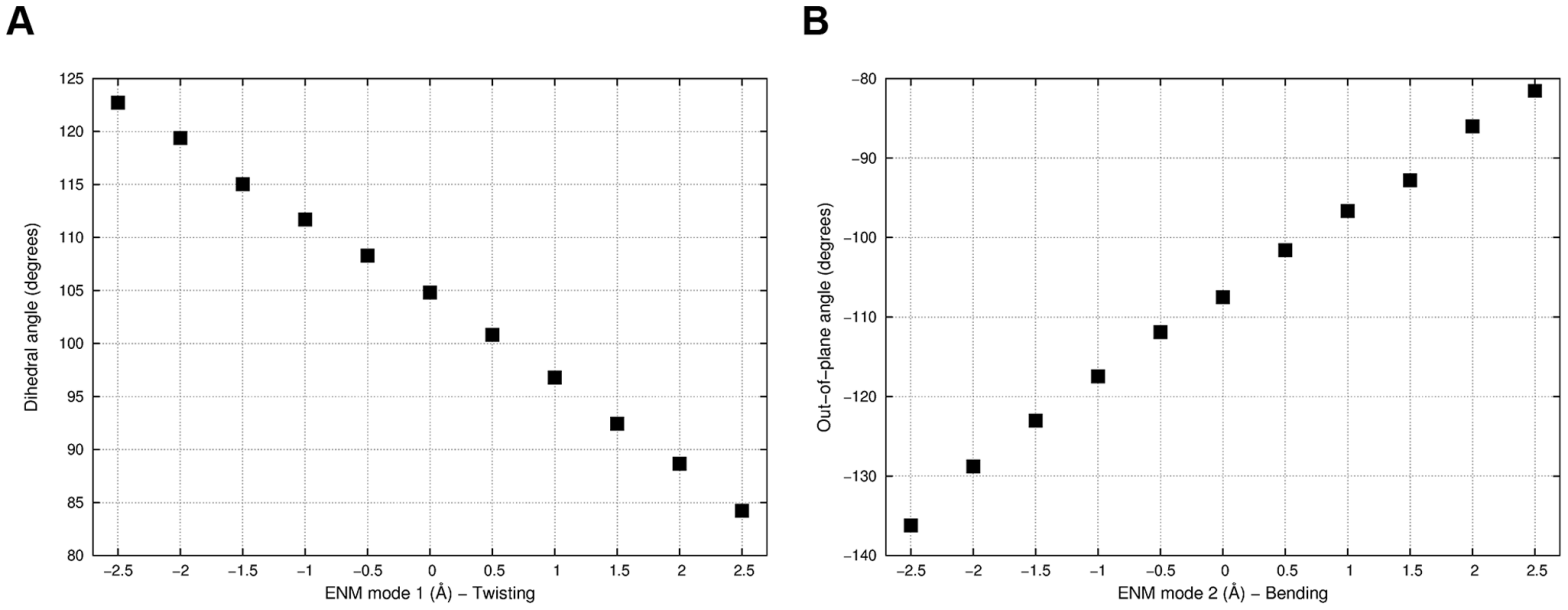
β-sheet structural changes. Representation of the twisting and bending modes of motions as dihedral angle (Thr243– His248– Glu320– Val325 Cα atoms) and out-of-plane bending angle (Thr243– Ile337– Val340– Glu320 Cα atoms) plotted along ENM modes 1 and 2, respectively.

**Figure 9:**
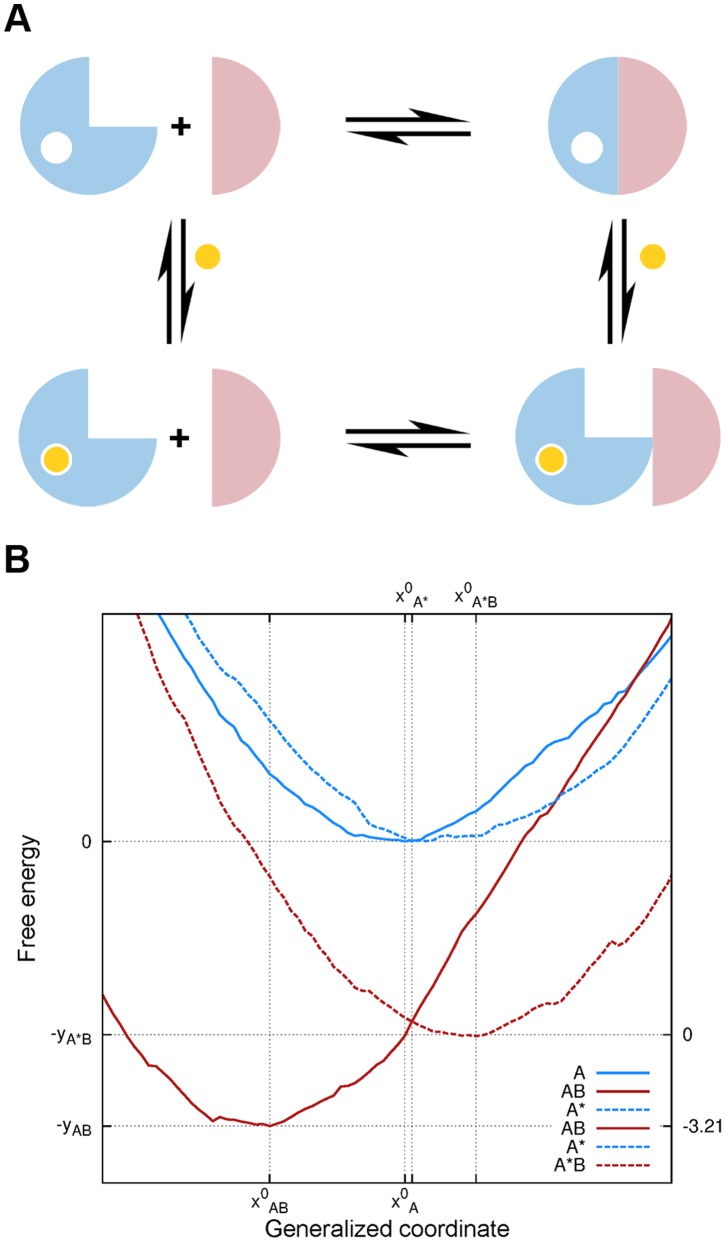
Model of binding. A. Schematic drawing of the proposed model of binding. B. Projection of the minimum free energy surface calculated with the biased MD simulation along the “twisting” β-sheet coordinate. On the left *y*-axis, the ΔΔG_bind_ value is reported in kcal mol^−1^.

To provide a better understanding of the structural changes between systems, the average twisting dihedral and out-of-plane bending of the β-sheet surface monitored during US simulations was projected along ENM mode 1 and 2, respectively (Figure 8). As a matter of fact, *A* is the only system preserving (on average) a β-sheet shape consistent with crystallographic structures (dihedral angle of 105 degrees and out-ot-plane angle of about −105 degrees). Differently, system *AB* significantly drifted towards negative values of the twisting coordinate (corresponding to a “flattening” of the surface of about 6 degrees), whereas positive values of both twisting and bending (corresponding to a “swelling” of the surface of 3–4 degrees in both angles) were observed for A* and A*B with minor differences between the two. Again, we note that the small size scale of such differences was not surprising, as the β-sheet is a fairly rigid structure [57]. Indeed, even though the flexibility is expected to be greater for parallel than antiparallel β-sheets of the same size [44], and it has been reported to increase by reducing the number of strands [58], larger motions could not have been reasonably expected. What was indeed surprising, was to find a closer resemblance between β-sheets of systems *A* and A* than between systems *A* and *AB*. In other words, the apo-monomeric form of the protein is more similar in structure to the holo-monomer than to the apo-heterodimer, meaning that the HIF-2α cavity is pre-structured to allocate ligands. A flattening in the β-sheet surface of the apo-monomeric form therefore occurs upon heterodimerization, as a consequence of the PAS-B/PAS-B mutual adaptation. In this scenario, ligand binding seems to lock the HIF-2α β-sheets surface on a heterodimerization less competent shape that might explain the protein-protein disrupting effect. Notably, allostery is not strictly involved in this model of binding, i.e. instead of inducing a heterodimerization unfavorable conformational change, disrupting ligands do hamper a favorable HIF-2α PAS-B/HIF-1β PAS-B mutual adaptation (Figure 9A).

To gain insight on the free energy changes upon binding, we projected the minimum free energy landscapes obtained through US simulations along the twisting coordinate (Figure 9B) Concerning the heterodimerization of the apo form, we estimate a relaxation free energy of about 3.5 kcal mol^−1^, meaning that, the strain in the β-sheet surface caused by HIF-1β binding is compensated by at least this energy amount arising from favorable mutual interactions. Unexpectedly, however, a relaxation process seems also to be involved for the holo-heterodimerization. This latter behavior is difficult to rationalize, and since the effect is only slightly apparent from the plot, we cannot rule out the possibility of artifacts. Indeed, the plot reported in Figure 9B has to be taken as a semi-quantitative description of binding, as more rigorous approaches (but also computationally much more expensive) should be employed whether a precise estimate of the absolute binding free energy is needed [59]. In spite of this, by taking the energy difference between the *AB* and A*B minima, we quantified the disrupting effect of compound **32** to be of the order of 3–4 kcal mol^−1^ (ΔΔG_bind_), which is consistent with an increase in the heterodimerization dissociation constant of about 3 order of magnitudes compared to an ordinary affinity ligand binding.

## Conclusions

Characterizing and predicting allosteric effects is one of the ultimate goals in biophysics, and Molecular Dynamics simulations can contribute in detecting and rationalizing the mechanism upon which proteins exhibit conformational changes in response to perturbations such as binding events. When dealing with allostery, one is usually concerned with either some global changes in conformation or in the transmission of such changes at distal sites form the origin of the perturbation. Here, we showed an example where local and extremely subtle changes in protein conformation upon binding are likewise challenging to be addressed and explained.

In this study, with the aim to characterize the allosteric mechanism at the basis of the ligand-induced HIF-2α PAS-B/HIF-1β PAS-B disruption, we discovered several evidences supporting a possible alternate interpretation of the accepted model of binding. According to our calculations, which consisted of both biased and unbiased MD simulations, the HIF-2α PAS-B domain appeared as a tightly structured protein which is unlikely to undergo large conformational motions in a submicrosecond time scale. We demonstrated that this behavior is consistent with a dynamically hydrated internal cavity, and we highlighted protein functional motions that might be exploited upon ligand recognition. Furthermore, we also showed that the HIF-2α β-sheet surface involved in the protein-protein interaction is able to adapt its shape in response to the presence of ligands inside the cavity or to the HIF-1β PAS-B domain. Not only we characterized this behavior from a structural point of view, but we also attempted to derive a semi-quantitative mechanistic model to describe the energetics of binding. As a result, we suggest a model of binding where ligands lock the HIF-2α β-sheet surface in a conformation less suited to optimally adapt to the HIF-1β counterpart. In this context, the protein-protein disruption is not properly referable to allostery, since the effect of the investigated ligand is to prevent a possible protein conformational change rather than inducing it. The discrepancies between our results and previous work might be found in the different starting structures employed in MD simulations. Indeed, we based our calculations on crystallographic coordinates, whereas previous work was performed starting from NMR derived data.

A striking feature of binding highlighted by our model, is that compound **32** only slightly alters the shape of the β-sheet surface, and in this respect it mostly acts as a “passive” disrupting ligand. We speculate that purposely designed bulkier ligands would be able to strain the β-sheet surface in an effective way so as to enhance the protein-protein disrupting effect, and, by doing so, to actually function as allosteric inhibitors. From this standpoint, Figure 9B depicts an intriguing scenario were relatively small ligand-induced perturbations on the β-sheet might result in an even more pronounced disrupting effect (greater ΔΔG_bind_). In prospect, the configurations obtained by biased MD simulations would be instrumental for structure-based drug design in pursuing an induced-fit β-sheet strain that would eventually lead to more potent PAS-B/PAS-B inhibitors.

## Supporting Information

File S1 The Supporting Information File S1 contains: **Figure S1.** Compound 32 and the holo-heterodimeric complex. **Figure S2.** Definition of the internal pocket. **Figure S3.** Elastic network model. **Figure S4.** Dynamic energy landscape model. **Figure S5.** RMSD of atomic positions. **Figure S6.** Structural features. **Figure S7.** Convergence of sampling. **Figure S8.** Water occupancy. **Figure S9.** Free energy along the twisting and bending coordinates. **Figure S10.** Free energy error estimation.
(DOCX)
