# Supplementary material for: Protein Dynamics of the HIF-2α PAS-B Domain upon Heterodimerization and Ligand Binding
Source: PLoS One. 2014 Apr 15;9(4):e94986. doi: 10.1371/journal.pone.0094986 (PMC3988133; doi:10.1371/journal.pone.0094986)
Supplement: File S1 — The Supporting Information File S1 contains: Figure S1. Compound 32 and the holo-heterodimeric complex. Figure S2. Definition of the internal pocket. Figure S3. Elastic network model. Figure S4. Dynamic energy landscape model. Figure S5. RMSD of atomic positions. Figure S6. Structural features. Figure S7. Convergence of sampling. Figure S8. Water occupancy. Figure S9. Free energy along the twisting and bending coordinates. Figure S10. Free energy error estimation. (DOCX) [file pone.0094986.s001.docx]

Supporting Information

Protein dynamics of the HIF-2α PAS-B domain upon heterodimerization and ligand binding

Matteo Masetti^*^, Federico Falchi ^*^, and Maurizio Recanatini

Department of Pharmacy and Biotechnology, Alma Mater Studiorum – Università di Bologna, via Belmeloro 6, 40126 Bologna, Italy.

^*^E-mail: [matteo.masetti4@unibo.it](mailto:matteo.masetti4@unibo.it) (MM)

**Content:**

Figure S1: Compound 32 and the holo-heterodimeric complex S3

Figure S2: Definition of the internal pocket S4

Figure S3: Elastic network model S5

Figure S4: Dynamic energy landscape model S6

Figure S5: RMSD of atomic positions S7

Figure S6: Structural features S8

Figure S7: Convergence of sampling S9

Figure S8: Water occupancy S10

Figure S9: Free energy along the twisting and bending coordinates S11

Figure S10: Free energy error estimation S12


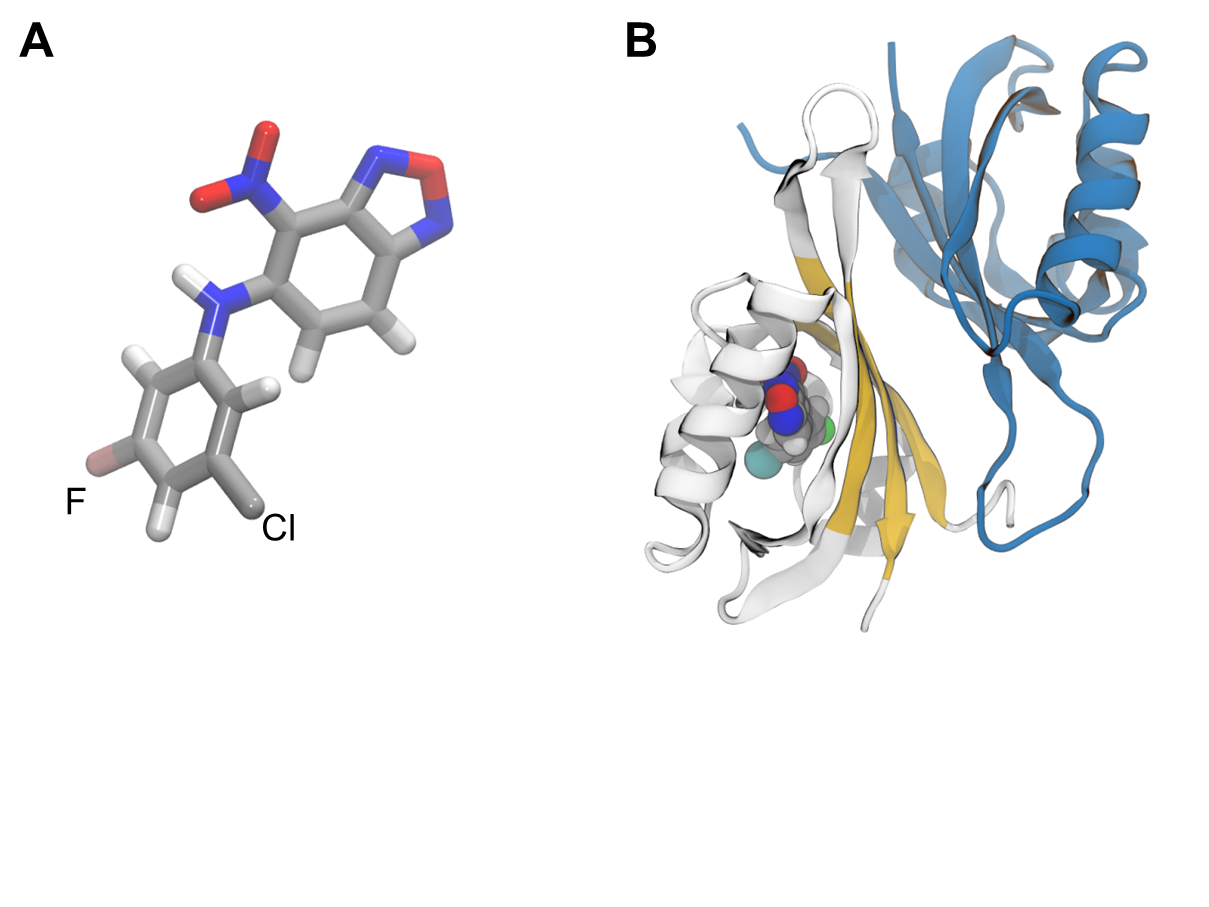


**Figure S1.** **Compound 32 and the holo-heterodimeric complex.** A. Chemical structure of compound 32. B. The ligand-bound HIF-2α PAS-B/HIF-1β PAS-B complex (system *A*B*).


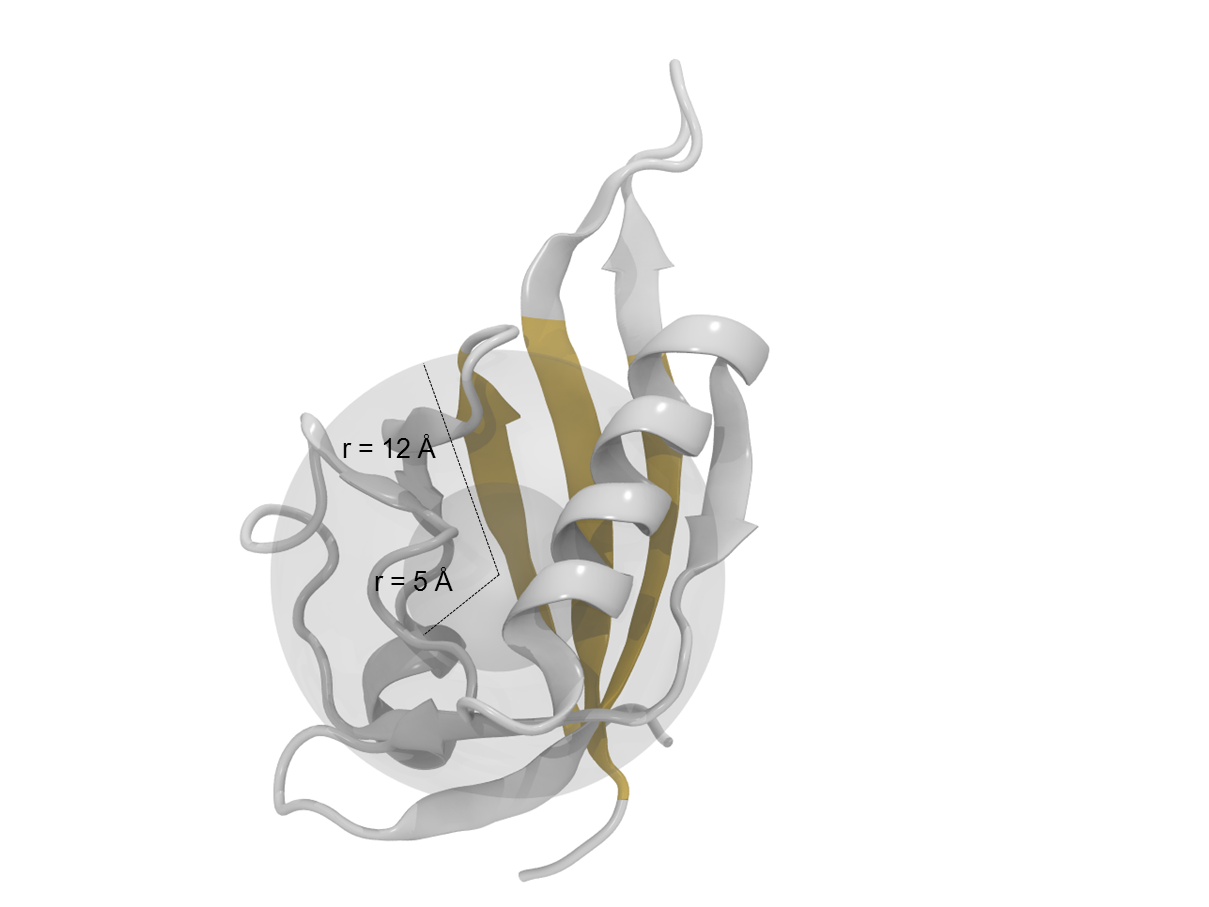


**Figure S2.** **Definition of the internal pocket.** The concentric spheres describing the HIF-2α PAS-B domain internal pocket and their position on the protein frame are shown.


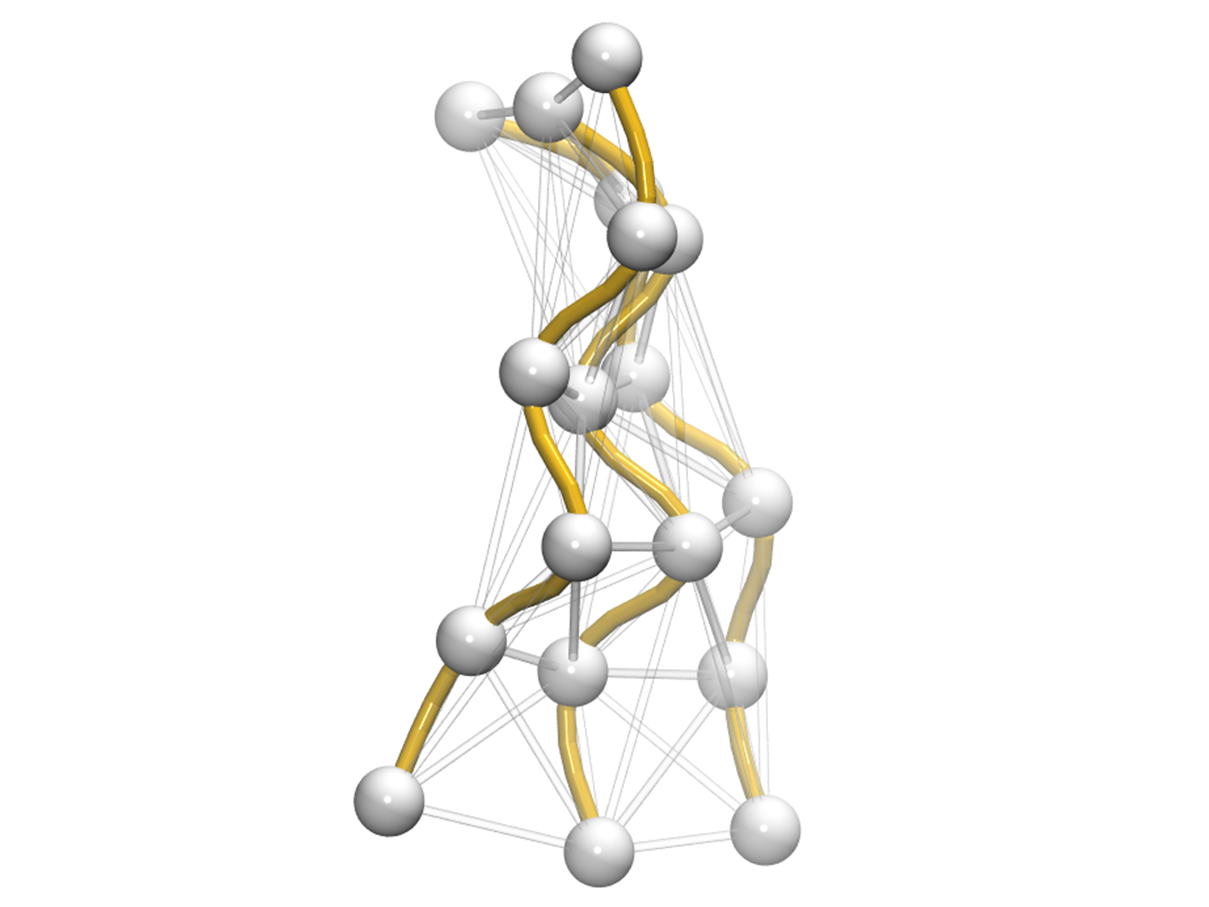


**Figure S3.** **Elastic network model.** Graphical representation of the elastic network model built to describe the flexibility of the HIF-2α PAS-B β-sheet.


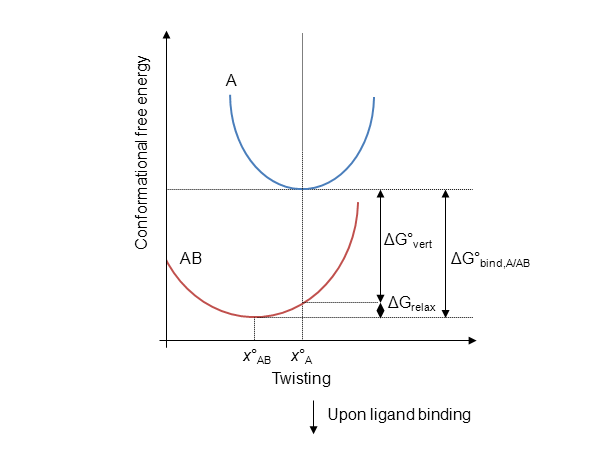

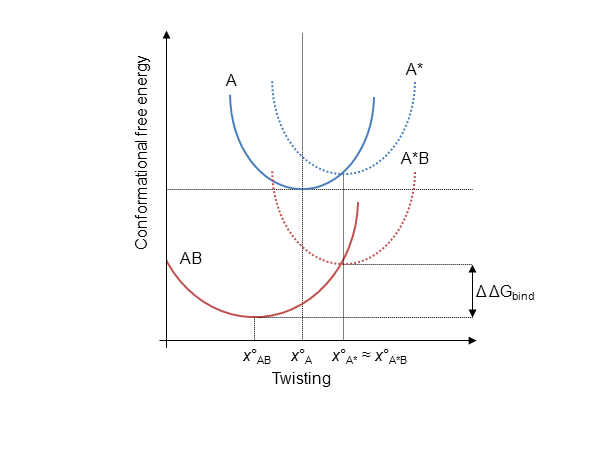


**Figure S4.** **Dynamic energy landscape model.** Schemes showing the approximated model used to assess the change in protein-protein free energy upon ligand binding. In the upper panel, the relative position of the free energy curves for systems *A* and *AB* is shown along with a graphical representation of the free energy contributions ΔG°_vert_ and ΔG_relax_. Upon binding (lower panel), the location of the *A** and *A*B* curves is shown relatively to the apo form surfaces. For simplicity, the *A** and *A*B* curves are shown as having the same shape and position of the minimum along the “twisting” axis (*x*^0^*_A*_* ≈ *x*^0^*_A*B_*). The real case is shown in Figure 9B, and commented in the main text.


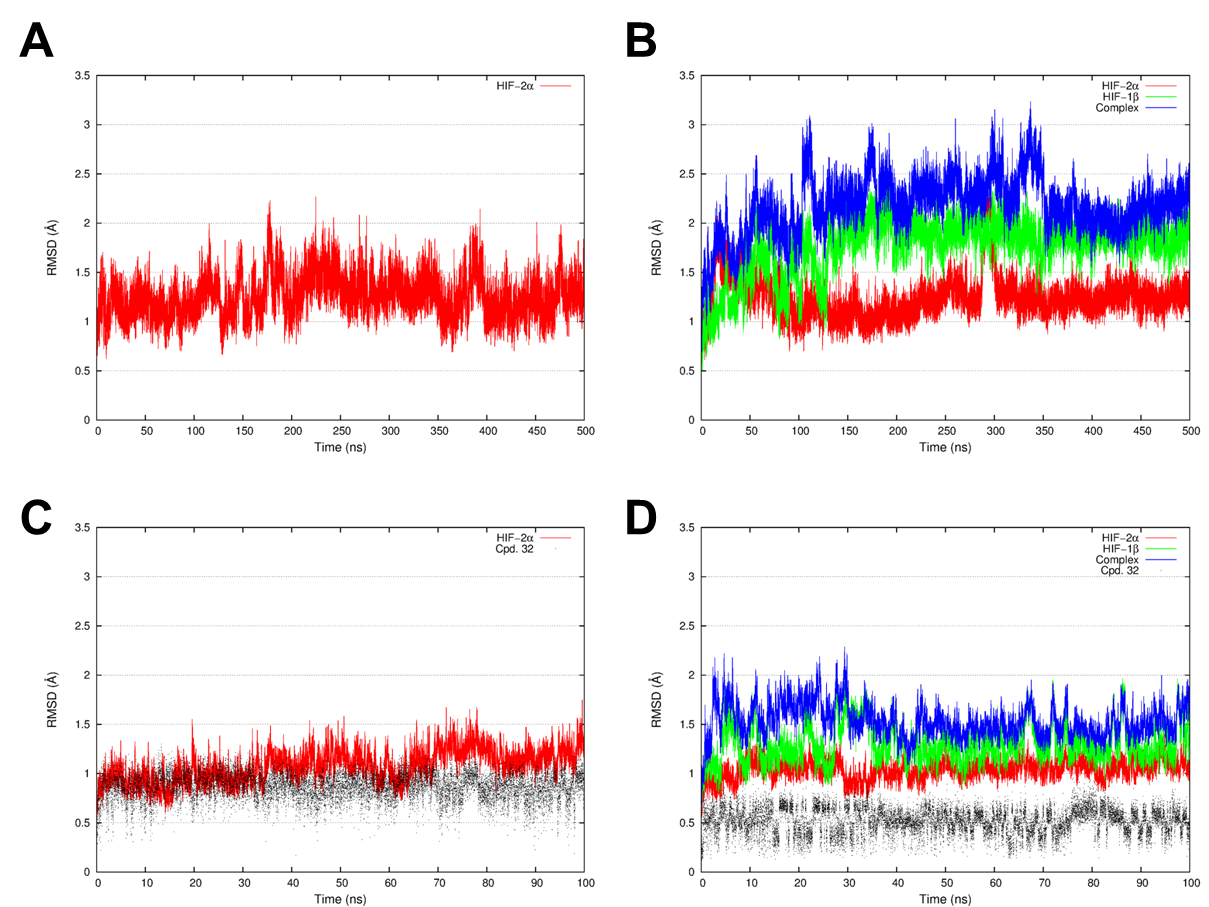


**Figure S5.** **RMSD of atomic positions.** ~~Structural stability of~~ Atomic RMSD over time calculated separately for each component of the simulated systems (*A* in panel A, *AB*: panel B, *A**: panel C, and *A*B*: panel D). Cα RMSD is shown as red, green, and blue lines for HIF-2α, HIF-1β, and the whole complex, respectively. In panels C and D, the RMSD calculated over all atoms of compound **32** is also shown as black dots.


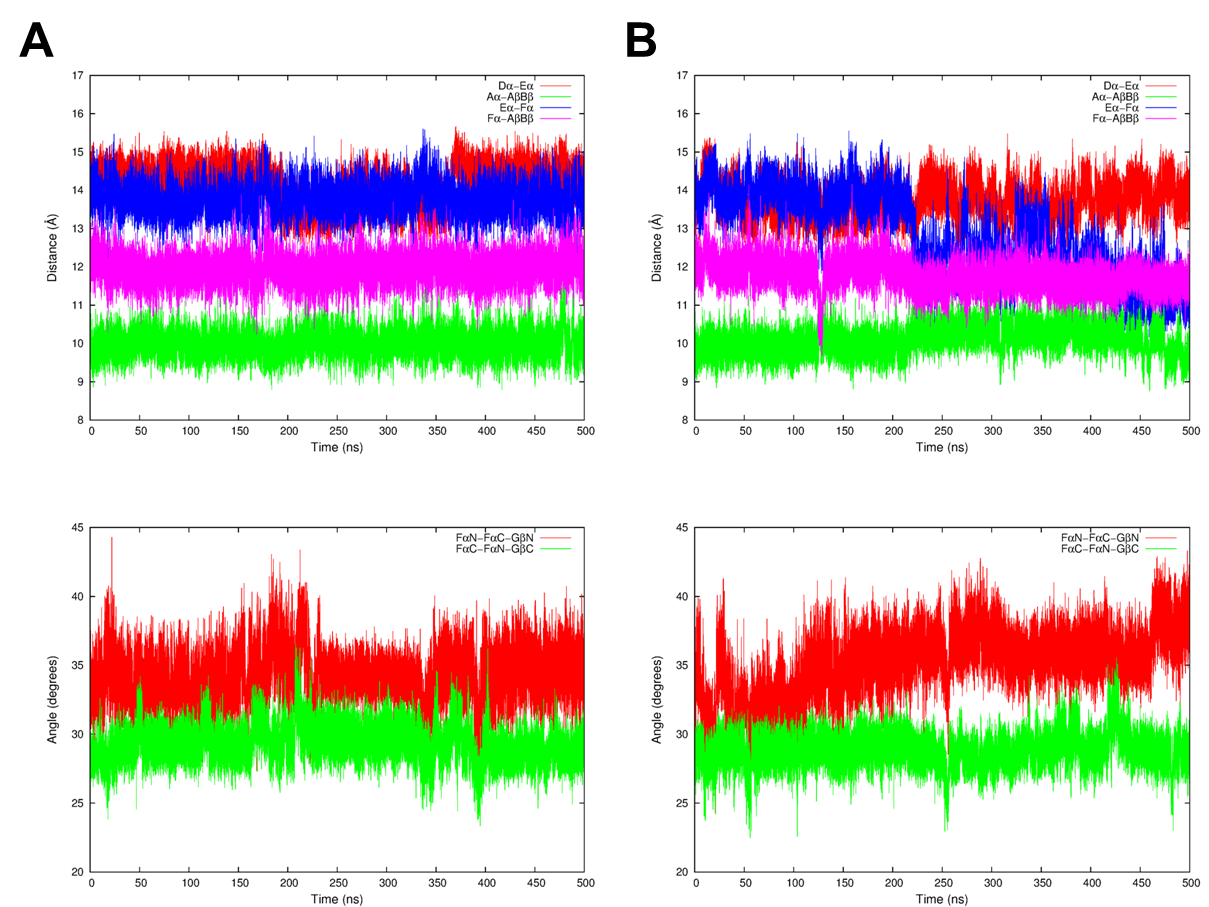


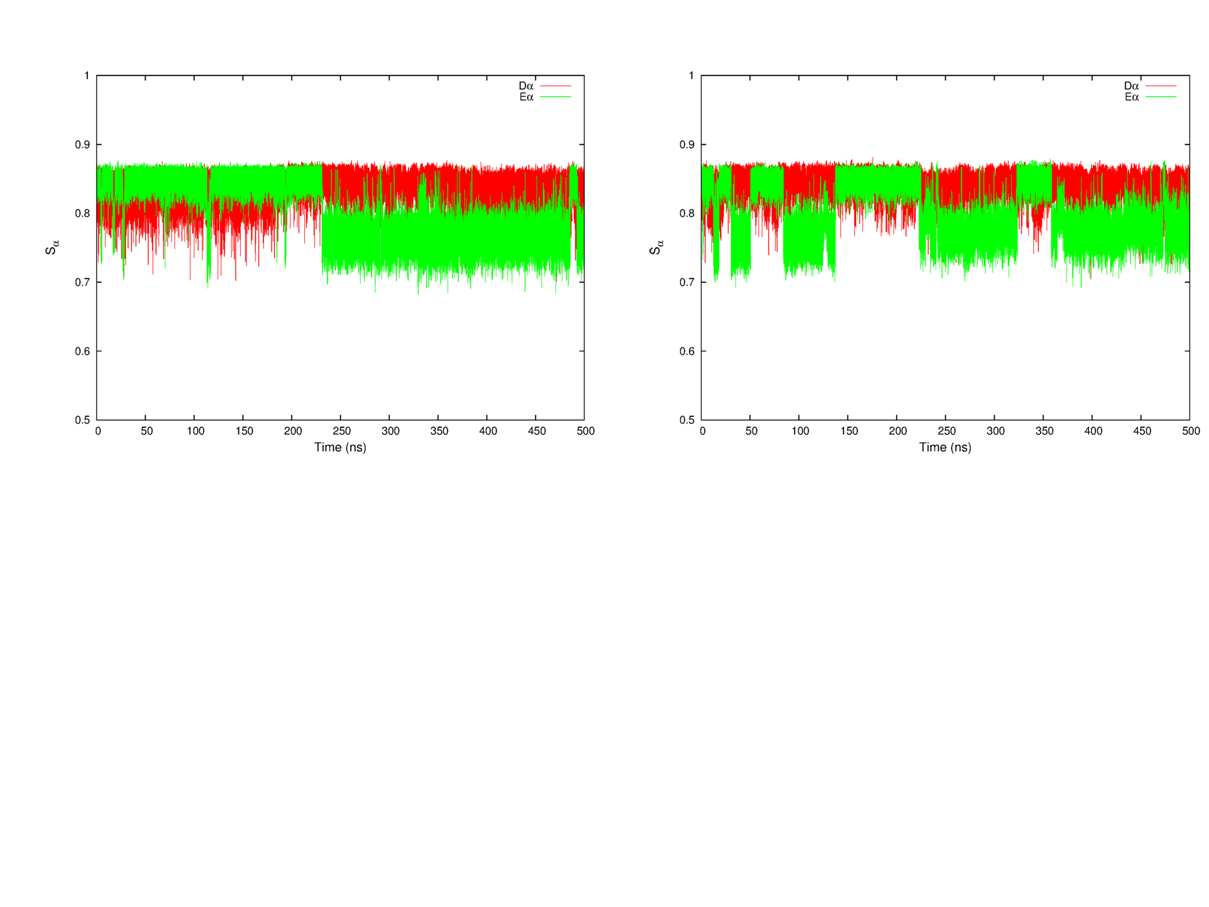


**Figure S6.** **Structural features.** Comparison of peculiar structural features for the *A* (panel A) and *AB* (panel B) systems over time. In particular, critical distances between structured portions in the front of the HIF-2α PAS-B domain (top) and angles between N- and C-termini between the Fα helix and Gβ strand (middle) are shown. In the bottom of the Figure, the folding degree of helices Dα and Eα is also shown (1 = completely folded helix).


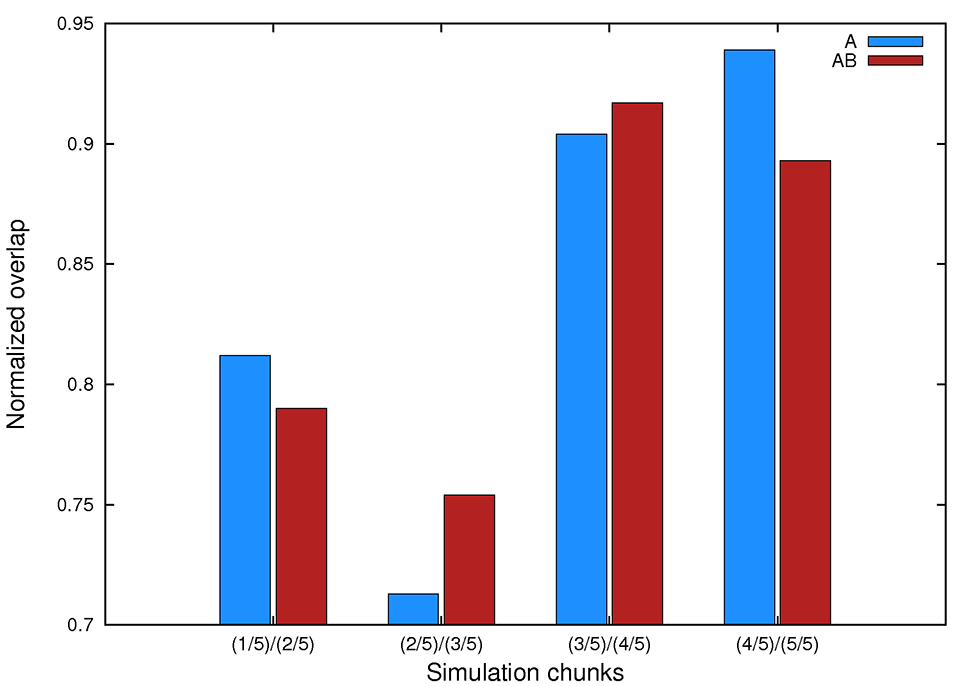


**Figure S7.** **Convergence of sampling.** Pairwise normalized overlap calculated for five trajectory chunks in the *A* and *AB* systems.


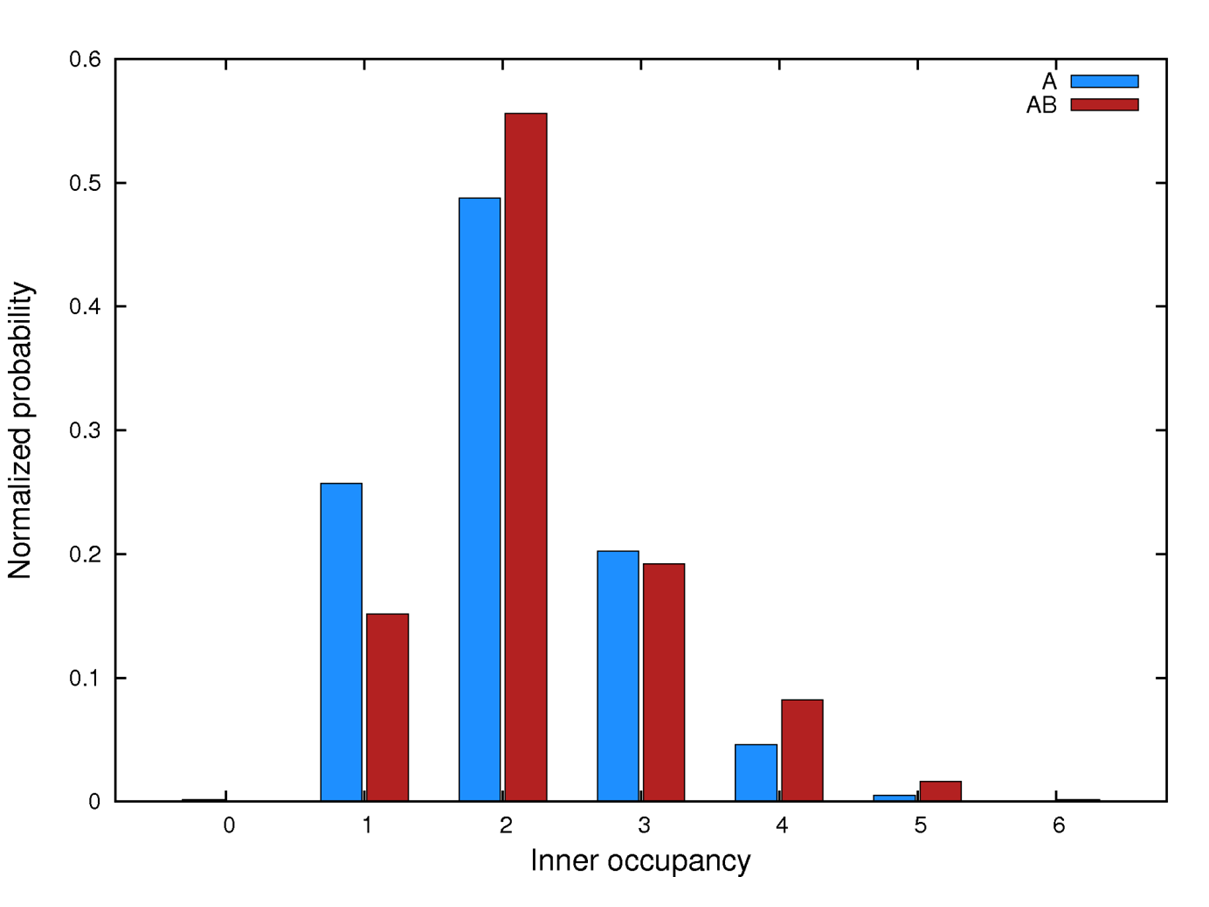


**Figure S8.** **Water occupancy.** The histogram shows the occupancy of the internal cavity for systems *A* and *AB*.


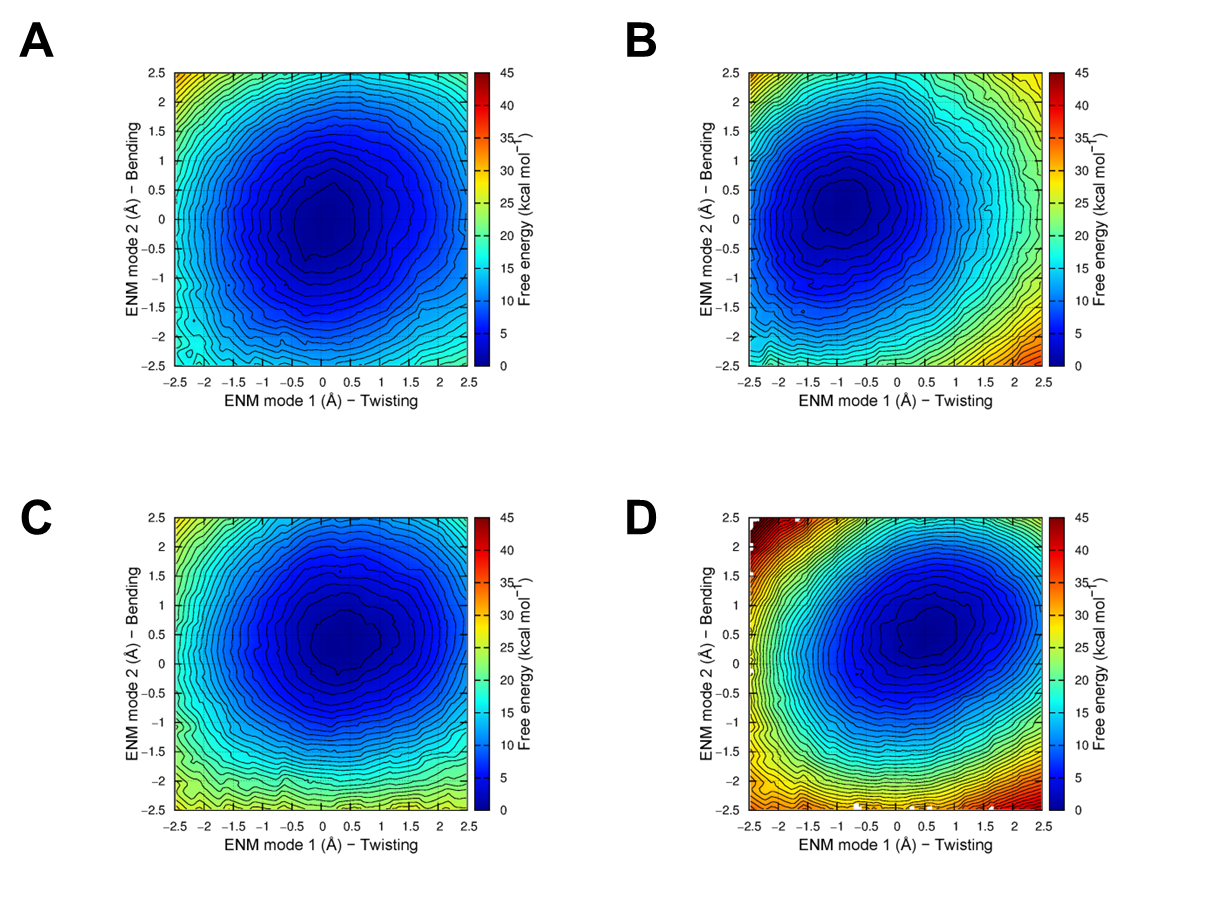


**Figure S9.** **Free energy along the twisting and bending coordinate.** Two dimensional free energy surface calculated with the umbrella sampling method (system *A* in panel A, *AB*: panel B, *A**: panel C, and *A*B*: panel D).


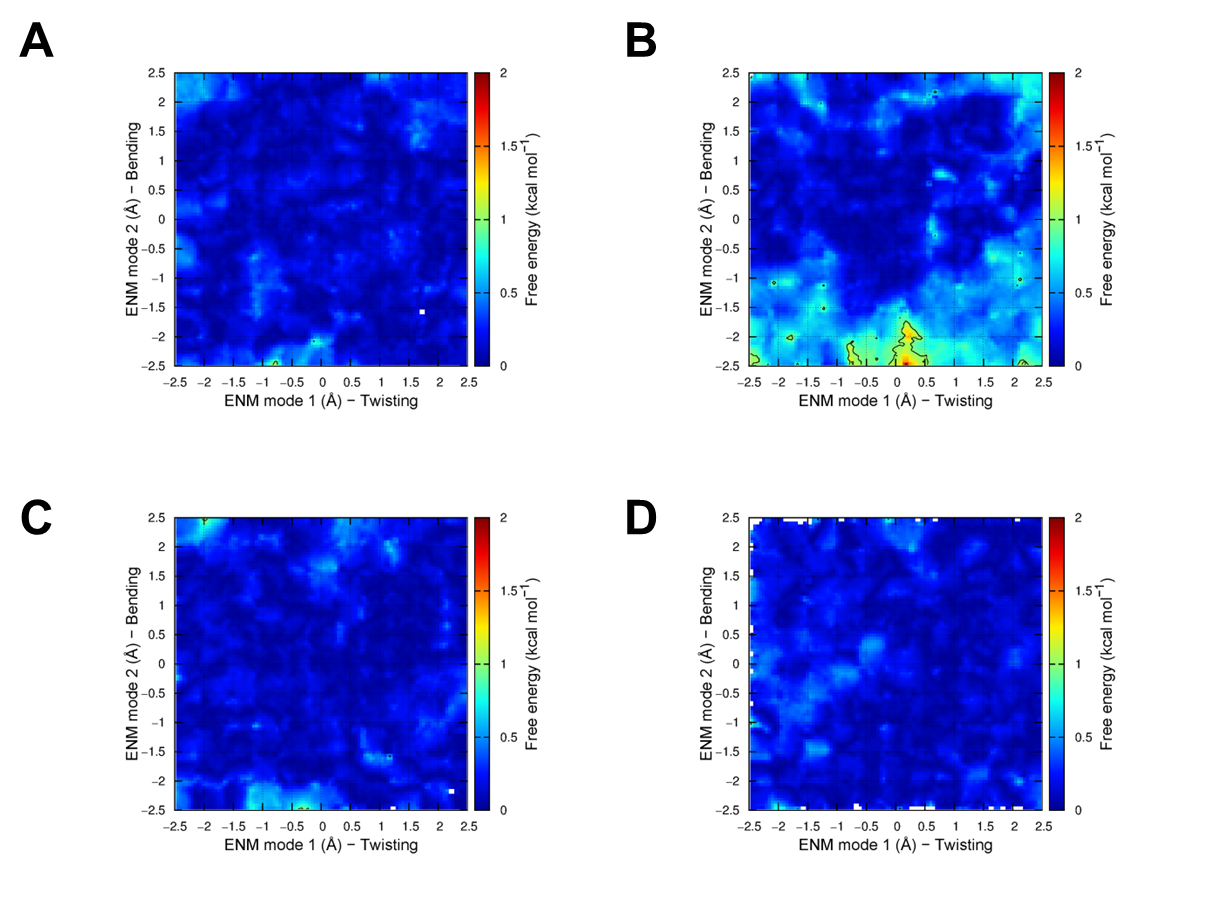


**Figure S10.** **Free energy error estimation.** Free energy difference calculated between the first and second half of the umbrella sampling simulations (system *A* in panel A, *AB*: panel B, *A**: panel C, and *A*B*: panel D).
